# Longitudinal reference data for the German D-AMI in major depression and healthy controls: implications for future ECT research

**DOI:** 10.3389/fpsyt.2026.1967204

**Published:** 2026-10-13

**Authors:** Sarah Kayser, Christian Plewnia, Magdalena Adan, Ruby Schaaf, Andreas J. Fallgatter

**Affiliations:** 1 Department of Psychiatry and Psychotherapy, University of Tübingen, Tübingen, Germany; 2 German Center for Mental Health (DZPG), partner site Tübingen, Tübingen, Germany; 3 Department of Psychiatry 3, Rheinhessen-Fachklinik Alzey, Alzey, Germany; 4 German Center for Neurodegenerative Disorders (DZNE), Tübingen, Germany

**Keywords:** autobiographical memory, autobiographical memory interview, cognitive side effects, electroconvulsive therapy, major depressive disorder, psychometric validation, retrieval consistency, test-retest variability

## Abstract

**Background:**

Electroconvulsive therapy (ECT) is an effective treatment for major depressive disorder (MDD). Retrograde autobiographical memory impairment is considered one of the clinically relevant adverse effects of ECT; however, no validated German instrument is available to assess autobiographical memory. Although longitudinal autobiographical memory data are available from previous studies of patients with depression not receiving ECT, corresponding reference data for the German-language AMI-SF have not been established. This study evaluated the feasibility and interrater reliability of the German Autobiographical Memory Interview-Short Form (D-AMI) and established longitudinal reference data in MDD and healthy controls.

**Methods:**

Sixty participants (30 patients with MDD and 30 healthy controls), none of whom received ECT during the observation period, completed the D-AMI at baseline and after a 6-week interval. Feasibility was assessed by completion rates and missing data. Interrater reliability was evaluated in a subset (n = 10) using a two-way random-effects intraclass correlation coefficient (ICC). Longitudinal performance was quantified using D-AMI score change and its baseline-normalized expression, retrieval consistency. Associations between autobiographical memory measures and depressive symptom severity were explored.

**Results:**

The D-AMI demonstrated a 100% completion rate with no missing data. Interrater reliability was ICC[2,1] = 0.71 (95% CI = 0.17-0.92), indicating considerable uncertainty. The magnitude of longitudinal inconsistency was greater in patients with MDD than in healthy controls, although this between-group difference should be interpreted cautiously because study site and rater were confounded with group.

**Conclusions:**

The D-AMI is a feasible instrument for assessing autobiographical memory in German-speaking populations, while the preliminary interrater reliability estimate requires confirmation in larger samples. The observed magnitude of longitudinal D-AMI inconsistency provides reference information for repeated assessments in the absence of ECT; however, the between-group difference requires cautious interpretation because group, site, and rater effects could not be separated. These findings provide the first longitudinal reference data for the German D-AMI and indicate that changes in autobiographical memory scores should not automatically be interpreted as evidence of ECT-related memory impairment. Rather, such changes should be considered in the context of test-retest variability and retrieval consistency.

## Introduction

Electroconvulsive therapy (ECT) is one of the most effective treatments for major depressive disorder (MDD) (1, 2). Its rapid and robust antidepressant effects make it a critical intervention in cases of high symptom burden, suicidality, or insufficient response to pharmacological treatments (3). Despite its well-established efficacy, concerns regarding cognitive side effects remain a central issue in both clinical practice and patient perception (4, 5).

ECT-related cognitive side effects may affect several domains, including postictal disorientation, attention and executive functioning, as well as anterograde memory (4, 6). These effects are typically transient and can be assessed using established instruments (4). In contrast, retrograde autobiographical memory impairment may persist beyond the acute treatment phase and is often considered the most distressing and clinically relevant cognitive adverse effect (6, 7). Difficulties recalling personally meaningful autobiographical memories have been reported following ECT and may contribute to disruptions in identity and continuity of self (8, 9).

Despite this clinical relevance, structured assessment of autobiographical memory in German-speaking populations remains limited, and no systematically evaluated German-language version of the Autobiographical Memory Interview-Short Form (AMI-SF) is currently available. Although the AMI-SF has predominantly been used in English-language research, adaptations into other languages have been reported, including a Polish adaptation and, more recently, a systematically evaluated Dutch translation (10, 11). Longitudinal autobiographical memory outcomes have previously been investigated in patients with depression not receiving ECT, including early studies with depressed comparison groups and more recent controlled studies comparing ECT with non-ECT treatments (12–16). However, corresponding longitudinal reference data for the German-language AMI-SF are not available. Together, these limitations represent an important methodological gap in the assessment and interpretation of ECT-related autobiographical memory outcomes.

The AMI-SF is one of the most widely used instruments for assessing autobiographical memory (17). It assesses consistency in the recall of autobiographical information across repeated assessments and has been widely applied in ECT research (18, 19). Interpretation of longitudinal AMI score changes, however, remains challenging because autobiographical memory retrieval is inherently reconstructive, and repeated assessments may show variability over time, even in the absence of ECT (5, 20, 21). Importantly, depression-related alterations in autobiographical memory may involve specific retrieval processes rather than a generalized reduction in autobiographical memory performance. Neuroimaging studies have identified altered engagement of neural systems supporting autobiographical retrieval in MDD, including regions of the default mode and salience networks involved in self-referential, mnemonic, affective, and cognitive-control processes (22, 23). Such alterations may affect qualitative or dynamic aspects of autobiographical retrieval that are not necessarily apparent from performance at a single assessment. Accordingly, longitudinal reference data from relevant clinical and healthy comparison groups are important for interpreting autobiographical memory outcomes (5). However, although longitudinal AMI-SF data are available from previous English-language studies, no German-language version of the AMI-SF has been systematically evaluated, and longitudinal reference data for repeated assessments with a German-language version have not yet been established.

The present study therefore aimed to evaluate the feasibility and interrater reliability of the German version of the AMI-SF (D-AMI) and to establish longitudinal reference data from repeated D-AMI assessments in patients with major depressive disorder and healthy controls. By characterizing test-retest variability and retrieval consistency in the absence of ECT, this study provides German-language longitudinal reference data for interpreting D-AMI outcomes in future ECT research. More broadly, the findings contribute to a more nuanced understanding of longitudinal autobiographical memory assessment and may help distinguish clinically meaningful memory changes from expected variability over repeated testing.

## Methods

### Study design and participants

This study employed a prospective cohort design to evaluate the D-AMI. The study, including both patients with MDD and healthy controls, was prospectively registered at ClinicalTrials.gov (NCT04516863). A total of 60 participants were included, comprising 30 patients with major depressive disorder and 30 healthy control participants. Healthy controls were recruited with the aim of achieving broadly similar age and sex distributions between groups. No individual matching or formal frequency matching was performed. Premorbid verbal intelligence was additionally considered during recruitment; however, exact matching on this variable was not achieved. Eligibility for the healthy control group required participants to be psychiatrically healthy based on a clinical psychiatric assessment. Thus, participants with a current psychiatric disorder were not eligible for inclusion. Healthy controls did not undergo a structured diagnostic interview; psychiatric history was assessed by self-report during the clinical interview. Although a history of depression was not specified as a separate formal exclusion criterion, none of the included healthy controls had a lifetime history of a depressive episode.

Eligibility required fulfillment of ICD-10 and DSM-5 diagnostic criteria for major depression and a HAM-D-17 score ≥17. Bipolar disorder was a predefined exclusion criterion. In patients, the Mini International Neuropsychiatric Interview (MINI) was administered to assess psychiatric comorbidities and relevant exclusion criteria. Other psychiatric comorbidities were permitted unless they constituted a predefined exclusion criterion. Patients were recruited between February 2021 and February 2022 from inpatient, day-clinic, and outpatient psychiatric services at the Rheinhessen-Fachklinik Alzey and assessed by a trained doctoral researcher. Healthy controls were recruited via advertisement between June and December 2025 and assessed at the University of Tübingen by a different trained doctoral researcher. Assessors were not blinded to participant group. Exclusion criteria for all participants included neurological disorders, severe cognitive impairment, substance use disorders, and insufficient German language proficiency. None of the participants received ECT, repetitive transcranial magnetic stimulation (rTMS), magnetic seizure therapy (MST), or any other neuromodulation treatment during the observation period.

The study protocol was approved by the local institutional ethics committees (Mainz 202014966; Tuebingen 041/2025BO2) and all procedures were conducted in accordance with the Declaration of Helsinki in its latest version. Written informed consent was obtained from all participants.

The study was designed to assess the feasibility, interrater reliability, and longitudinal performance of the D-AMI while establishing reference data for repeated assessments in the absence of ECT.

### Study protocol

All participants underwent two assessment time points: a baseline assessment (T1) and a follow-up assessment scheduled 6 weeks later (T2; allowable window: ± 3 days). At both time points, the D-AMI was administered to assess autobiographical memory consistency over time. In the patient group, depressive symptom severity and global clinical status were additionally reassessed at follow-up. Patients received ongoing clinical treatment during the 6-week observation period. Information on the number of antidepressant medications was available at both assessment time points; however, detailed information on individual agents, dosages, and other treatment modifications was not systematically available.

The 6-week interval was chosen to allow assessment of longitudinal changes in autobiographical memory and to provide reference data for repeated D-AMI assessments in the absence of ECT while remaining clinically feasible.

### Clinical and neuropsychological assessment

At baseline, all participants underwent standardized clinical and neuropsychological assessment. Depressive symptom severity was assessed using the clinician-rated 17-item Hamilton Depression Rating Scale (HAM-D-17) (24) and the self-report Inventory of Depressive Symptomatology (IDS-SR) (25).

Global cognitive functioning was screened using the Mini-Mental State Examination (MMSE) (26), and premorbid verbal intelligence was estimated using the Multiple Word Test - B (MWT-B) (27). Subjective global memory change was assessed using the Global Self-Evaluation of Memory (GSE-My) (28). The GSE-My was originally developed to assess the perceived global impact of ECT on memory. In the present study, it was used as a treatment-independent global rating of perceived memory change rather than specifically in relation to ECT. At follow-up (T2), participants rated their perceived memory change over the 6-week period since the baseline assessment (T1). The GSE-My uses a 7-point Likert-type scale ranging from 1 (marked worsening) to 7 (marked improvement), with 4 indicating no perceived change. In addition, sociodemographic and clinical variables were recorded.

### Autobiographical memory assessment

Autobiographical memory was assessed using the D-AMI, a structured interview designed to assess consistency in the recall of autobiographical information across repeated assessments.

The original AMI-SF (17) was translated into German using a standardized forward-backward translation procedure (29). The instrument was first translated into German by a professional translation service and subsequently back-translated into English by an independent translator. Discrepancies were reviewed and resolved to ensure conceptual equivalence. The German adaptation preserved the six-section item structure and baseline-anchored scoring framework of the original AMI-SF. Adaptations primarily concerned German-language wording, treatment-related terminology, and the assessment schedule. Because the D-AMI was developed for use across neuromodulation settings, including ECT and rTMS, the administration manual contains treatment-specific references to both modalities. In the present study, D-AMI assessments were performed in patients with MDD and healthy controls at baseline and after 6 weeks. The complete German D-AMI as administered, together with the interviewer instructions and baseline and follow-up scoring rules, is provided as **Supplementary Material**.

At baseline (T1), participants provided autobiographical information for each item. At follow-up (T2), the same items were reassessed without participants having access to their prior responses. Responses were evaluated in relation to the autobiographical information provided at baseline; additional information newly reported at follow-up was not counted as an improvement in consistency. If a participant initially reported a different event or person at follow-up, the interviewer redirected the participant to the event or person identified at baseline. Items that had received a score of 0 at baseline or had not been administered at baseline were omitted from follow-up scoring, in accordance with the original AMI-SF scoring instructions. Follow-up scoring was therefore strictly anchored to the information provided at baseline. A score of 2 was assigned for fully consistent recall, 1 for partially consistent recall, and 0 for inconsistent or absent recall. Partial-credit scoring followed the item-category-specific criteria specified in the scoring instructions. Total scores were calculated by summing item-level ratings.

Following the traditional AMI-SF scoring approach (17), retrieval consistency was calculated for each participant as the ratio of the D-AMI total score at follow-up (T2) to the D-AMI total score at baseline (T1), expressed as a percentage: Retrieval consistency (%) = (D-AMI total score at T2/D-AMI total score at T1) × 100. Higher percentages indicate greater consistency of autobiographical information across assessments. Because newly reported information at follow-up does not increase the score beyond information available at baseline, follow-up scores are structurally bounded by baseline scores. Accordingly, longitudinal analyses focus on the magnitude of score inconsistency and between-group differences rather than on the direction of within-group change itself.

Interpretation of autobiographical memory performance was based on changes over time and comparison with longitudinal changes observed in healthy controls, rather than fixed cut-off values.

### Outcome measures

Longitudinal D-AMI performance was characterized using absolute change in D-AMI total scores and retrieval consistency, with the latter representing a baseline-normalized expression of longitudinal score change rather than an independent outcome. Secondary outcome measures included depressive symptom severity, global cognitive functioning, and subjective global memory change.

### Statistical analysis

Statistical analyses were conducted using standard statistical software (SPSS). Descriptive statistics are reported as means and standard deviations.

Baseline group differences were assessed using independent-samples t-tests for continuous variables and chi-square tests for categorical variables.

Within-group longitudinal changes were summarized descriptively. Because the longitudinal scoring procedure structurally constrains follow-up scores not to exceed baseline scores, paired-samples tests were not interpreted as substantive tests of longitudinal change. Between-group differences in change scores and retrieval consistency were analyzed using independent-samples t-tests, with corresponding Cohen’s d effect sizes. Homogeneity of variance was assessed using Levene’s test, and Welch’s t-test was used when this assumption was violated.

Distributional characteristics of longitudinal D-AMI inconsistency and retrieval consistency were additionally summarized using ranges and the 25th, 50th, and 75th percentiles. Test–retest associations between baseline and follow-up D-AMI total scores were assessed separately for each group using Pearson correlation coefficients and absolute-agreement intraclass correlation coefficients (ICCs).

To examine differences between patients and healthy controls over time, a mixed-design analysis of variance (ANOVA) was conducted with time (baseline vs. follow-up) as the within-subject factor and group (patients vs. healthy controls) as the between-subject factor, including the group × time interaction. Effect sizes are reported as partial eta squared (ηp²).

To assess the potential influence of baseline differences in premorbid verbal intelligence and global cognitive performance, sensitivity analyses were conducted using linear regression models with D-AMI change scores and retrieval consistency as dependent variables and group, MWT-B score, and baseline MMSE score as predictors.

Exploratory Pearson correlation analyses were conducted within the patient group to examine associations between autobiographical memory measures and depressive symptom severity. These included correlations between baseline D-AMI scores and depression severity, as well as correlations between changes in autobiographical memory (ΔD-AMI) and changes in depressive symptoms (ΔHAM-D, ΔIDS-SR). Change scores were calculated as baseline minus follow-up values (T1 - T2), such that positive ΔD-AMI values indicate a decline in autobiographical memory performance over time.

Additional exploratory analyses examined associations of retrieval consistency with depressive symptom measures and the association between duration of the current depressive episode and longitudinal D-AMI change. Exploratory analyses additionally examined subjective global memory change assessed with the GSE-My. Follow-up ratings were summarized descriptively and categorized as perceived worsening (<4), no change (=4), or improvement (>4). Within the MDD group, associations of GSE-My ratings with D-AMI retrieval consistency, longitudinal D-AMI change, and depressive symptom improvement were examined using correlation analyses.

Interrater reliability was assessed in a subset of interviews (n = 10) using a two-way random-effects ICC with absolute agreement. ICC estimates are reported with 95% confidence intervals to reflect the precision of the estimate.

Assumptions of normality were assessed prior to analysis.

All statistical tests were two-tailed with a significance level set at p < 0.05.

## Results

A total of 60 participants were included, comprising 30 patients with major depressive disorder and 30 healthy controls. All 30 patients with MDD and all 30 healthy controls completed both baseline and follow-up assessments, with no dropouts between the two assessment time points.

### Sample characteristics

There were no significant differences between patients and healthy controls in age or sex distribution (**Table 1**). However, healthy controls showed significantly higher premorbid verbal intelligence (MWT-B) and higher baseline global cognitive performance (MMSE). Baseline D-AMI total scores did not differ significantly between patients with MDD and healthy controls (54.07 ± 3.22 vs. 54.40 ± 3.30, p = 0.693). The maximum possible baseline D-AMI score was 60. Baseline scores ranged from 48 to 60 in the MDD group and from 48 to 58 in healthy controls. One patient with MDD (3.3%) and no healthy control reached the maximum score; 5 patients with MDD (16.7%) and 8 healthy controls (26.7%) scored ≥58.

**Table 1:** Baseline demographic, clinical, cognitive, and autobiographical memory characteristics of patients with major depressive disorder and healthy controls.

Characteristic	Patients (n=30)	Healthy controls (n=30)	P
Age, years, mean (SD), range	44.33 (17.71), 18–74	41.90 (20.26), 18–80	0.622
Female, n (%)	20 (66.7)	20 (66.7)	1.000
MWT-B score	26.13 (4.53)	30.70 (2.93)	<0.001
MMSE at baseline	28.83 (1.21)	29.77 (0.50)	<0.001
D-AMI total at baseline	54.07 (3.22)	54.40 (3.30)	0.693
HAM-D-17 at baseline	20.93 (2.53)	—	—
IDS-SR at baseline	43.47 (10.55)	—	—
Age at onset of depression, years	35.20 (18.50)	—	—
Duration of current depressive episode, months	11.27 (11.31)	—	—
Number of depressive episodes	3.17 (2.20)	—	—
Psychiatric comorbidity, n (%)	21 (70.0)	—	—
Anxiety disorder, n (%)	17 (56.7)	—	—
Post-traumatic stress disorder, n (%)	3 (10.0)	—	—
Obsessive-compulsive disorder, n (%)	1 (3.3)	—	—

Values are presented as mean (standard deviation) unless otherwise indicated. Group differences were analyzed using independent-samples t-tests and chi-square tests where appropriate. Clinical characteristics specific to major depressive disorder are reported for the patient group only. MWT-B, Multiple Word Test-B; MMSE, Mini-Mental State Examination; D-AMI, German Autobiographical Memory Interview-Short Form; HAM-D-17, 17-item Hamilton Depression Rating Scale; IDS-SR, Inventory of Depressive Symptomatology-Self-Report.

In the MDD group, the mean age at onset of depression was 35.20 years (SD = 18.50). The mean duration of the current depressive episode was 11.27 months (SD = 11.31), with a median of 9 months (IQR = 5.25-12) and a range of 1–48 months. Additional clinical characteristics, including baseline depressive symptom severity, number of depressive episodes, and psychiatric comorbidities, are summarized in **Table 1**.

Educational attainment was recorded as highest school-leaving qualification and vocational/professional training rather than as years of education. A university entrance qualification was reported by 11 patients with MDD (36.7%) and 18 healthy controls (60.0%), and completed vocational/professional training by 17 patients (56.7%) and 26 healthy controls (86.7%).

### Longitudinal changes in autobiographical memory

In the patient group, mean D-AMI total scores changed from 54.07 (SD = 3.22) at baseline to 47.67 (SD = 6.17) at follow-up, corresponding to a mean longitudinal inconsistency of 6.40 points (SD = 5.82; 95% CI = 4.23-8.57). In healthy controls, scores changed from 54.40 (SD = 3.30) to 52.03 (SD = 3.89), corresponding to a mean inconsistency of 2.37 points (SD = 1.56; 95% CI = 1.78-2.95). Because the longitudinal scoring procedure constrains follow-up scores not to exceed baseline scores, the direction of within-group change is inherent to the scoring approach. The informative finding was the between-group difference in the magnitude of longitudinal inconsistency, reflected by a significant group × time interaction, F(1,58) = 13.43, p < 0.001, ηp² = 0.188, with greater longitudinal inconsistency in patients with MDD than in healthy controls (**Table 2**).

**Table 2:** Longitudinal changes in D-AMI total scores over the 6-week follow-up period.

Group	Baseline mean (SD)	Follow-up mean (SD)	Mean longitudinal inconsistency (SD)	95% CI of mean longitudinal inconsistency
Patients	54.07 (3.22)	47.67 (6.17)	6.40 (5.82)	4.23-8.57
Healthy controls	54.40 (3.30)	52.03 (3.89)	2.37 (1.56)	1.78-2.95

D-AMI total scores at baseline and follow-up in patients with major depressive disorder and healthy controls. Values are presented as mean (standard deviation), with 95% confidence intervals provided for mean longitudinal inconsistency. Positive values indicate lower D-AMI performance at follow-up relative to baseline.

The distribution of longitudinal D-AMI inconsistency was broader in patients with MDD than in healthy controls. In the MDD group, inconsistency ranged from 0 to 27 points, with a 25th percentile of 3.25, a median of 5.00, and a 75th percentile of 8.00. In healthy controls, inconsistency ranged from 0 to 5 points, with corresponding percentiles of 1.25, 2.00, and 3.75.

In a sensitivity analysis adjusting for baseline MWT-B and MMSE scores, group remained significantly associated with longitudinal D-AMI change (B = 4.45, SE = 1.40, 95% CI = 1.65-7.25, t(56) = 3.18, p = 0.002), indicating that the between-group difference in the magnitude of longitudinal D-AMI inconsistency remained significant after adjustment for baseline differences in premorbid verbal intelligence and global cognitive performance.

### Retrieval consistency

Retrieval consistency was 88.24% (SD = 10.76) in patients with MDD and 95.60% (SD = 2.96) in healthy controls. As homogeneity of variance was violated (Levene’s F(1,58) = 11.24, p = 0.001), Welch’s t-test was used and confirmed significantly lower retrieval consistency in the MDD group, t(33.37) = -3.61, p = 0.001, Cohen’s d = -0.93. This group difference also remained significant after adjustment for baseline MWT-B and MMSE scores (B = -8.02, SE = 2.59, 95% CI = -13.21 to -2.82, t(56) = -3.09, p = 0.003). These findings provide longitudinal reference data on retrieval consistency in repeated D-AMI assessments in the absence of ECT. Longitudinal D-AMI scores for both groups are illustrated in **Figure 1**.

**Figure 1:**
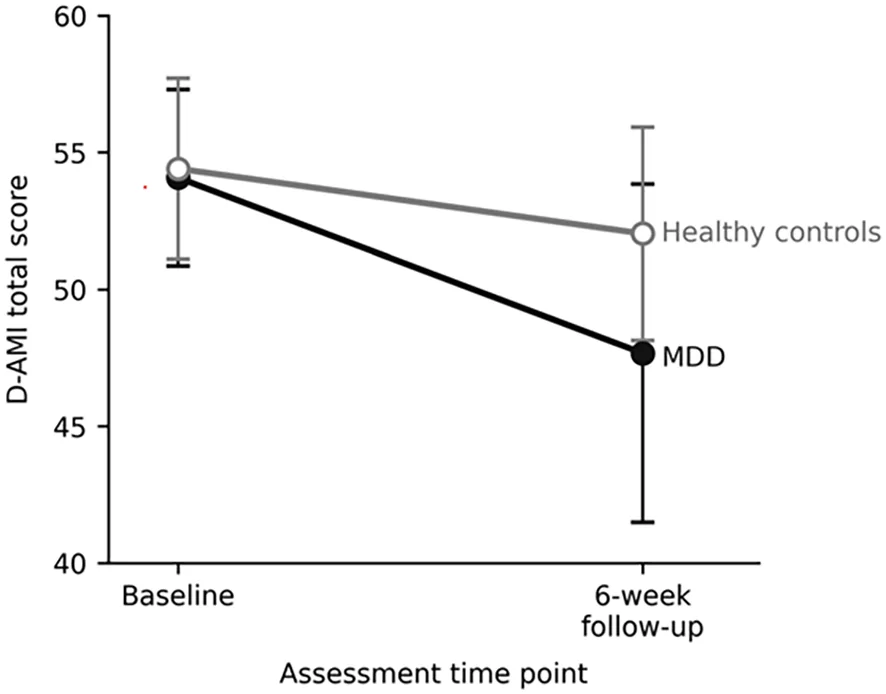
Longitudinal changes in D-AMI scores in patients with major depressive disorder and healthy controls.

Retrieval consistency ranged from 48.08% to 100% in patients with MDD, with a 25th percentile of 84.62%, a median of 90.37%, and a 75th percentile of 93.59%. In healthy controls, retrieval consistency ranged from 90.00% to 100%, with corresponding percentiles of 93.27%, 96.23%, and 97.75%. Baseline and follow-up D-AMI total scores were correlated at r = 0.366 (p = 0.047) in patients with MDD and r = 0.918 (p < 0.001) in healthy controls. The corresponding test–retest ICCs were 0.165 in patients with MDD and 0.747 in healthy controls.

Mean total scores on the German Autobiographical Memory Interview-Short Form (D-AMI) at baseline and follow-up assessments in patients with major depressive disorder (MDD) and healthy controls. The magnitude of longitudinal D-AMI score inconsistency over the 6-week interval was greater in patients with MDD than in healthy controls. Error bars represent standard deviations (SDs).

### Depressive symptom course and associations with autobiographical memory

Depressive symptom severity decreased significantly over the 6-week follow-up period. At baseline, HAM-D-17 scores had a mean of 20.93 (SD = 2.53), a median of 21 (IQR = 19–23), and ranged from 17 to 25. Mean HAM-D-17 scores decreased to 12.23 (SD = 5.22) at follow-up, corresponding to a mean reduction of 8.70 points (95% CI = 6.60–10.80), t(29) = 8.46, p < 0.001, Cohen’s dz = 1.54. Similarly, mean IDS-SR scores decreased from 43.47 (SD = 10.55) to 32.37 (SD = 11.00), corresponding to a mean reduction of 11.10 points (95% CI = 8.10–14.10), t(29) = 7.56, p < 0.001, Cohen’s dz = 1.38.

Baseline D-AMI scores were not significantly associated with baseline depressive symptom severity (HAM-D-17: r = -0.084, p = 0.658; IDS-SR: r = -0.186, p = 0.325). Furthermore, changes in D-AMI scores were not significantly associated with changes in depressive symptom severity over the 6-week follow-up period (ΔHAM-D-17: r = 0.222, p = 0.237; ΔIDS-SR: r = 0.209, p = 0.268). Retrieval consistency was also not significantly associated with baseline depressive symptom severity (r = 0.19, p = 0.30) or with clinical improvement over time (r = -0.23, p = 0.22). Duration of the current depressive episode was not significantly associated with longitudinal D-AMI change (r = 0.271, p = 0.148).

The number of antidepressant medications remained unchanged between baseline and follow-up in 26 of 30 patients (86.7%), whereas four patients (13.3%) showed a reduction from two antidepressant medications to one. No patient showed an increase in the number of antidepressant medications. In a sensitivity analysis restricted to patients with an unchanged number of antidepressant medications (n = 26), the mean longitudinal D-AMI inconsistency was 5.04 points (SD = 3.82).

### Subjective memory change

At follow-up, healthy controls uniformly reported no subjective change in memory on the GSE-My (all ratings = 4). In patients with MDD, the mean GSE-My score was 4.70 (SD = 0.84; median = 5). Overall, 16 patients (53.3%) reported subjective memory improvement (GSE-My >4), 13 (43.3%) reported no change (=4), and one (3.3%) reported worsening (<4). Thus, despite the greater longitudinal D-AMI inconsistency observed in the MDD group, subjective global memory change was predominantly rated as stable or improved.

Within the MDD group, GSE-My ratings were not significantly associated with D-AMI retrieval consistency (r = 0.036, p = 0.851) or longitudinal D-AMI inconsistency (r = -0.024, p = 0.900). Higher GSE-My ratings were associated with greater improvement in self-reported depressive symptoms (IDS-SR: r = 0.435, p = 0.016), whereas the association with clinician-rated symptom improvement did not reach statistical significance (HAM-D-17: r = 0.295, p = 0.114).

### Feasibility and reliability

The D-AMI was successfully administered in all participants at both assessment time points, resulting in a completion rate of 100% with no missing data, indicating good feasibility in both patient and control groups.

Interrater reliability, assessed in a subset of interviews (n = 10), yielded an ICC[2,1] of 0.71 (95% CI = 0.17-0.92, p = 0.009). The wide confidence interval indicates considerable uncertainty around this estimate.

## Discussion

The present study provides evidence for the feasibility of the German D-AMI and a preliminary estimate of its interrater reliability. The instrument was completed by all participants at both time points with no missing data, indicating good feasibility in both clinical and non-clinical populations. The interrater reliability estimate was ICC[2,1] = 0.71; however, the wide confidence interval and small subsample require cautious interpretation.

These findings provide initial support for the feasibility of the German D-AMI and preliminary information on its interrater reliability in clinical and research settings. Beyond this initial psychometric evaluation, the present findings provide longitudinal reference data on repeated D-AMI assessments in the absence of ECT. The magnitude of longitudinal D-AMI inconsistency over the 6-week interval was greater in patients with MDD than in healthy controls, as indicated by a significant group-by-time interaction. Because the longitudinal scoring procedure structurally constrains follow-up scores not to exceed baseline scores, the direction of within-group change itself should not be interpreted as an independent empirical finding. Within the patient group, depressive symptoms improved substantially over the 6-week follow-up period; however, neither baseline D-AMI performance nor longitudinal changes in D-AMI scores were significantly associated with depressive symptom severity or its change over time. Retrieval consistency was relatively high in both groups but was significantly lower in patients with MDD than in healthy controls (88.24% vs. 95.60%). The distributional analyses further demonstrated greater interindividual variability in longitudinal D-AMI performance in patients with MDD, whereas changes in healthy controls were comparatively narrowly distributed. Because retrieval consistency is mathematically derived from baseline and follow-up D-AMI scores, D-AMI change and retrieval consistency should not be regarded as independent or mutually corroborating outcomes. Rather, retrieval consistency provides a baseline-normalized expression of the magnitude of longitudinal D-AMI inconsistency. These findings provide an important methodological context for interpreting autobiographical memory outcomes in future ECT studies.

The absence of a significant between-group difference in baseline D-AMI scores suggests that no difference in overall D-AMI performance was detectable between patients with MDD and healthy controls at the initial assessment. Although only one participant reached the maximum possible score, baseline scores were concentrated in the upper part of the scale in both groups. A pronounced ceiling effect is therefore unlikely, but some upper-range compression cannot be excluded and may have reduced the ability of the D-AMI total score to detect between-group differences at baseline. In addition, depression-related alterations in autobiographical memory may affect specific aspects of autobiographical memory rather than result in a generalized reduction in overall performance. Thus, comparable overall D-AMI scores at baseline do not exclude more specific alterations in autobiographical memory performance.

The longitudinal findings add an important perspective to this baseline observation. Because follow-up D-AMI performance is evaluated in relation to the autobiographical information provided at baseline, it reflects the consistency with which previously available information can subsequently be retrieved. Although the groups showed comparable overall D-AMI performance at baseline, patients with MDD subsequently showed greater longitudinal D-AMI inconsistency than healthy controls. The group difference in the present study therefore became apparent primarily in the longitudinal stability of autobiographical retrieval rather than in overall performance at the initial assessment.

This pattern may be considered in the context of neurobiological evidence indicating altered recruitment of neural systems supporting autobiographical retrieval in depression. Neuroimaging studies particularly implicate regions within the default mode and salience networks involved in self-referential, mnemonic, affective, and cognitive-control processes (22, 23). Such alterations could conceivably affect qualitative or dynamic aspects of autobiographical retrieval without necessarily producing a generalized reduction in overall performance at a single assessment.

The present finding of comparable baseline D-AMI scores but lower longitudinal retrieval consistency in MDD is compatible with this possibility. Depressive symptoms improved substantially during follow-up, whereas the exploratory analyses did not identify statistically significant associations between depressive symptom severity and D-AMI performance. However, these null findings should be interpreted cautiously given the restricted baseline HAM-D-17 range, modest sample size, and exploratory nature and number of correlation analyses, and therefore do not establish that D-AMI performance is independent of depressive symptom severity. However, because the present study did not assess neural activity or the specific cognitive processes underlying autobiographical retrieval, this neurobiological interpretation remains hypothesis-generating. The mechanisms underlying the lower longitudinal stability in the MDD group therefore warrant further investigation.

Previous ECT research using the AMI and related autobiographical memory measures has consistently reported reductions in autobiographical memory performance following treatment, particularly after bilateral ECT (7, 18, 19, 30). Interpretation of these findings, however, remains challenging because autobiographical memory retrieval is inherently reconstructive and repeated assessments are associated with some degree of variability even in the absence of ECT (5, 20, 21). An additional distinction emerged between objective longitudinal D-AMI performance and subjective memory appraisal. Whereas patients with MDD showed greater longitudinal D-AMI inconsistency than healthy controls, their subjective global memory ratings were predominantly unchanged or improved, and GSE-My ratings were not associated with longitudinal D-AMI inconsistency. Instead, higher subjective memory ratings were associated with greater improvement in self-reported depressive symptoms. This dissociation suggests that subjective and objectively assessed memory change may capture partly distinct aspects of cognitive functioning. Previous ECT research has likewise shown that subjective memory assessments may diverge from objective cognitive measures; notably, Berman et al. (28) reported an association between GSE-My ratings and autobiographical memory impairment following ECT. The absence of such an association in the present non-ECT sample further underscores the importance of appropriate reference data when interpreting autobiographical memory outcomes following ECT.

Longitudinal autobiographical memory changes in depressed patients not receiving ECT have been examined for several decades, including early studies with depressed non-ECT comparison groups (12). In a randomized comparison of right unilateral ECT and algorithm-based pharmacological treatment in treatment-resistant bipolar depression, retrieval consistency following the acute treatment period was 72.9% following ECT and 80.8% following pharmacological treatment (14). At 6-month follow-up, corresponding consistency rates were 64.3% and 72.3%, respectively (15). More recently, Jelovac et al. (16) likewise reported longitudinal autobiographical memory inconsistency in depressed patients not receiving ECT. Together, these studies demonstrate that incomplete consistency across repeated autobiographical memory assessments also occurs in the absence of ECT. Nwatarali et al. (11) recently evaluated a Dutch translation of the CUAMI-SF in patients with depression and healthy controls, providing longitudinal data from a translated version of the instrument. The retrieval consistency observed in the present MDD sample (88.24%) was higher than that reported in these previous depressed comparison groups, although direct numerical comparisons should be made cautiously because of differences in patient populations, treatment settings, follow-up intervals, and study designs. The greater longitudinal inconsistency observed in the present sample may therefore reflect depression-related differences in autobiographical memory retrieval; however, the present exploratory analyses do not allow a more specific attribution of this difference to depressive symptom severity.

The present findings provide longitudinal reference data on the magnitude of D-AMI score inconsistency across repeated assessments without ECT exposure. Although the present study did not include patients undergoing ECT, these findings suggest that longitudinal changes in D-AMI performance observed in future ECT studies should be interpreted in the context of expected test–retest variability. Comparison with appropriate reference data and consideration of retrieval consistency may help distinguish clinically meaningful autobiographical memory alterations from expected longitudinal variability.

This pattern suggests that reductions in D-AMI total scores over time should not, in themselves, be interpreted as evidence of clinically meaningful autobiographical memory impairment. Such changes may reflect, at least in part, variability in autobiographical memory retrieval and the test-retest characteristics of the instrument. At the same time, retrieval consistency was significantly lower in patients with MDD than in healthy controls, indicating that longitudinal variability was more pronounced in the patient group. The present findings are consistent with Sackeim’s argument that autobiographical memory outcomes should not be interpreted solely on the basis of raw score changes but should be considered in relation to expected variability and retrieval consistency (5).

Autobiographical memory is inherently reconstructive and dynamic, and variations in detail, contextual framing, or retrieval strategies may lead to measurable changes in scores that do not necessarily reflect actual loss of memory content (20, 21). Accordingly, changes in raw D-AMI scores should be interpreted cautiously and should not, in isolation, be equated with clinically meaningful retrograde amnesia without considering retrieval consistency, longitudinal reference data, and the psychometric characteristics of the instrument (5).

These findings have important implications for the assessment of cognitive side effects following ECT and related convulsive therapies. Autobiographical memory disturbances are among the most clinically relevant cognitive concerns associated with ECT (6, 9). The present findings indicate that repeated D-AMI assessments may show measurable score reductions even in the absence of ECT, thereby providing an important methodological context for interpreting longitudinal autobiographical memory outcomes. Consequently, interpretation of longitudinal D-AMI assessments should consider retrieval consistency and longitudinal reference data rather than relying solely on absolute score changes. This underscores the need for careful interpretation of longitudinal memory assessments; retrieval consistency provides a baseline-normalized representation of longitudinal D-AMI change that may facilitate interpretation across repeated assessments (5).

Several limitations should be considered when interpreting the present findings. First, the sample size was modest, and the study was not designed to establish normative data or clinical cut-offs, which limits the generalizability of the findings. The duration of the current depressive episode varied considerably across patients (range 1–48 months), which may have contributed to heterogeneity in longitudinal autobiographical memory performance. Although episode duration was not significantly associated with D-AMI change in the present sample, the modest sample size limits the ability to exclude an influence of pre-baseline episode duration. Second, interrater reliability was assessed in a small subset of participants only, resulting in considerable uncertainty around the ICC estimate. Third, the study did not include patients undergoing ECT. Consequently, the clinical utility of the D-AMI for detecting ECT-associated autobiographical memory changes remains to be established. Conclusions regarding treatment-related effects therefore remain indirect. Although baseline D-AMI scores did not differ between groups, healthy controls showed higher premorbid verbal intelligence and baseline global cognitive performance. Importantly, however, the between-group differences in longitudinal D-AMI change and retrieval consistency remained significant in sensitivity analyses adjusting for MWT-B and baseline MMSE scores. Nevertheless, the baseline cognitive differences between groups should be considered when interpreting the findings. Furthermore, the present study was not designed to determine the mechanisms underlying the greater longitudinal D-AMI inconsistency observed in patients with MDD. Although no significant associations with depressive symptom severity were observed, the modest sample size and exploratory nature of these analyses preclude conclusions regarding the role of depression-related factors. Patients with MDD and healthy controls were recruited and assessed at different study sites by different trained doctoral researchers, who were not blinded to participant group. Because study site and rater were linked to participant group, potential site- or rater-related effects cannot be disentangled from group effects, and the absence of rater blinding may have introduced assessment bias.

In addition, patients received ongoing clinical treatment during the observation period. Although the number of antidepressant medications was available at both assessment time points and remained unchanged in most patients, detailed information on individual medications, dosages, and other treatment modifications was not systematically available. Consequently, potential effects of treatment on longitudinal autobiographical memory performance cannot be excluded.

Finally, the follow-up interval was limited to six weeks and therefore does not allow conclusions regarding the longer-term stability of repeated D-AMI assessments or longer-term changes in autobiographical memory.

Despite these limitations, the present study provides a methodological basis for future investigations of autobiographical memory following ECT. The longitudinal reference data generated in the absence of ECT provide a reference context for interpreting longitudinal autobiographical memory changes in future ECT studies. A key next step will be to apply the D-AMI in patients undergoing ECT to directly assess longitudinal autobiographical memory changes associated with treatment. Such studies will be essential to determine the sensitivity and clinical utility of the instrument in detecting potential ECT-associated memory alterations and to further clarify the relationship between D-AMI score changes and patient-reported cognitive outcomes (7, 8).

In conclusion, the German D-AMI was a feasible instrument for assessing autobiographical memory, with preliminary interrater reliability findings that require confirmation in larger samples. Beyond these psychometric properties, the present study provides longitudinal reference data for repeated D-AMI assessments in the absence of ECT. The findings indicate that longitudinal changes in D-AMI scores should be interpreted within the context of longitudinal reference data and retrieval consistency rather than relying on absolute score changes alone. The greater longitudinal inconsistency observed in patients with MDD provides preliminary evidence that longitudinal D-AMI performance may differ between clinical and non-clinical populations; however, this finding requires confirmation in study designs that separate group effects from site and rater effects. These findings provide a basis for future investigations of autobiographical memory following ECT.

## Data Availability

The data supporting the findings of this study are available from the corresponding author upon reasonable request.
