# Supplementary material for: Longitudinal reference data for the German D-AMI in major depression and healthy controls: implications for future ECT research
Source: Front Psychiatry. 2026 Oct 13;17:1967204. doi: 10.3389/fpsyt.2026.1967204 (PMC13710296; doi:10.3389/fpsyt.2026.1967204)
Supplement: Supplementary file 1 [file DataSheet1.pdf]

Supplementary Material

German Autobiographical Memory Interview–Short Form (D-AMI): Questionnaire, Administration Instructions, and Scoring Rules

Original instrument: McElhiney MC, Moody BJ, Sackeim HA. Autobiographical Memory Interview–Short Form (AMI-SF). Version 3, April 2001.

The German Autobiographical Memory Interview–Short Form (D-AMI) was developed as a German adaptation of the original English Autobiographical Memory Interview–Short Form (AMI-SF) for use in neuromodulation research, including electroconvulsive therapy (ECT) and repetitive transcranial magnetic stimulation (rTMS).

The following supplementary material provides the German-language D-AMI as administered in the present study, including the complete questionnaire, interviewer instructions, and baseline and follow-up scoring rules. The instrument is presented in German to preserve the wording of the version administered to study participants.

Accordingly, the original German administration manual contains legacy treatment-specific wording referring to both rTMS and ECT. These references reflect the broader intended use of the German adaptation and were not used to define the assessment schedule in the present study.

In the present study, none of the participants received ECT, rTMS, magnetic seizure therapy (MST), or any other neuromodulation treatment during the 6-week observation period, and the D-AMI was administered to patients with major depressive disorder (MDD) and healthy controls at baseline and after 6 weeks as described in the Methods.

The German adaptation preserved the six-section item structure and baseline-anchored scoring framework of the original English AMI-SF. At follow-up, responses were scored relative to the autobiographical information provided at baseline. Items scored 0 or not administered at baseline were omitted from follow-up scoring; newly reported information at follow-up did not receive additional credit; and partial-credit scores were assigned according to the item-category-specific scoring criteria reproduced below. Adaptations from the English version primarily concerned German-language wording, treatment-related terminology, and the assessment schedule.

Part A. D-AMI Questionnaire

Patient (Name)	ID	Geburtsjahr

FRAGEBOGEN ZUM AUTOBIOGRAFISCHEN GEDÄCHTNIS – DEUTSCHE KURZVERSION DES AUTOBIOGRAFISCHEN GEDÄCHTNIS INTERVIEW (D-AMI)

TEIL 1 – FAMILIENMITGLIED

Prä-Test<br>Datum: __/__/___	Post-Test<br>Datum: __/__/___	
Zur Einleitung sagen Sie bitte: **„Während diesem Interview, werde ich Sie darum bitten, sich an verschiedene Erlebnisse und Erfahrungen aus Ihrer Vergangenheit, zu erinnern. Ich werde Sie darum bitten, sich an sehr spezifische Details zu erinnern. Das Interview gibt uns die Möglichkeit ihre Fähigkeit sich an Details aus Ihrer Vergangenheit zu erinnern, einzuschätzen. Ähnliche Interviews kommen im weiteren Verlauf der Studie auf Sie zu.“**		
[Interviewer: Sie beginnen diesen Abschnitt, indem Sie den Befragten/ die Befragte bitten einen Verwandten/ eine Verwandte zu benennen. Zu dem/ der genannten Verwandten stellen Sie dem Patienten/ der Patientin fünf Fragen. Wenn mehrere Verwandte genannt werden, fragen Sie nach dem/ der Verwandten, mit dem der/ die Befragte den häufigsten Kontakt hat. Hat der/ die Befragte keine Verwandte, fragen Sie nach dem engsten/ der engsten Freund/-in).]		
	**Notizen**	**Summe**
**ICH WERDE IHNEN EINIGE FRAGEN ÜBER EIN FAMILIENMITGLIED STELLEN:<br>WIE HEISST DER/ DIE VERWANDTE, DER/ DIE IHNEN AM WICHTIGSTEN IST, DER/ DIE ABER NICHT MIT IHNEN ZUSAMMENLEBT MIT VOR- UND NACHNAMEN?**		
Prä-Test: Name		
[Interviewer: Wenn beim POST-Test ein anderer Name als bei dem Prä-Test genannt wird, erinnern Sie den Patienten/ die Patientin, bitte an den ursprünglich genannten Namen und die Beziehung und fahren mit Frage 1-5 fort.]		
Post-Test: Name		
**WIE IST SEINE/ IHRE BEZIEHUNG ZU IHNEN?**		
Prä-Test (nur): Beziehung		
**FRAGE 1: AN WELCHEM TAG HAT ER/ SIE GEBURTSTAG?**		
Prä-Test: Monat & Tag		
Post-Testung: Monat & Tag		
**FRAGE 2: WIE ALT WAR ER/SIE ZU DEM ZEITPUNKT, ALS SIE MIT DIESER STUDIE BEGONNEN HABEN?**		
Prä-Test: Alter		
Post-Testung: Alter		
**FRAGE 3: WIE LAUTETE DIE VOLLSTÄNDIGE ADRESSE VON IHM/ IHR, ZU DEM ZEITPUNKT, ALS SIE MIT DIESER STUDIE BEGONNEN HABEN?**		
Prä-Test: Adresse		

Post-Testung: Adresse		
**FRAGE 4: WIE LAUTETE SEINE/ IHRE TELEFONNUMMER EINSCHLIESSLICH VORWAHL ZU DEM ZEITPUNKT, ALS SIE MIT DIESER STUDIE BEGONNEN HABEN?**		

Prä-Test: Telefonnummer		
Post-Testung: Telefonnummer		
**FRAGE 5: WELCHE PERSON(EN) HAT/ HABEN ZU DEM ZEITPUNKT, ALS SIE MIT DIESER STUDIE BEGONNEN HABEN, MIT DEM/DER VERWANDTEN ZUSAMMENGELEBT?**		
Prä-Test: Mitbewohner/-in		
Post-Testung: Mitbewohner/-in		
	**Prä-Test: Summe**	
	**Post-Test: Summe**	

Patient (Name)	ID	Geburtsjahr

FRAGEBOGEN ZUM AUTOBIOGRAFISCHEN GEDÄCHTNIS – DEUTSCHE KURZVERSION DES AUTOBIOGRAFISCHEN GEDÄCHTNIS INTERVIEW (D-AMI)

TEIL 2 –REISE

Prä-Test<br>Datum: __/__/___	Post-Testung<br>Datum: __/__/___	
[Interviewer: Sie beginnen diesen Abschnitt, indem Sie den Befragten/ die Befragte darum bitten, sich an die LETZTE große Reise, die er/ sie vor der Untersuchung gemacht hat, zu erinnern. Wenn der/ die Befragte nie mehr als 100 Meilen, 160 Km verreist ist, fragen Sie nach der letzten Reise, mit Übernachtung außerhalb von Zuhause.]		
	**Notizen**	**Summe**
**ICH WERDE IHNEN EINIGE FRAGEN ÜBER DIE LETZTE GRÖSSERE REISE STELLEN, DIE SIE UNTERNOMMEN HABEN, BEVOR SIE MIT DIESER STUDIE BEGONNEN HABEN: WOHIN FÜHRTE SIE IHRE LETZTE REISE, BEI DER SIE MINDESTENS EINE NACHT MEHR ALS 150 KILOMETER VON ZUHAUSE ENTFERNT VERBRACHT HABEN?**		
Prä-Test: Reiseziel		
[Interviewer: Wenn beim POST-Test eine andere Reise als beim Prä-Test genannt wird, erinnern Sie den Patienten/die Patientin, bitte an die ursprünglich genannte Reise und fahren mit Frage 1-5 fort.]		
Post-Test: Reiseziel		
**IN WELCHEM JAHR UND WELCHEM MONAT HABEN SIE DIE REISE UNTERNOMMEN?**		
Prä-Test (nur): Monat & Jahr der Reise		
**FRAGE 1: WIE VIELE TAGE WAREN SIE EINSCHLIESSLICH AN- UND ABREISE UNTERWEGS?**		
Prä-Test: Anzahl der Reisetage		
Post-Testung: Anzahl der Reisetage		
**FRAGE 2: WIE HEISST DAS HOTEL (BZW. DIE PERSON), BEI DEM/DER SIE SICH DIE MEISTE ZEIT WÄHREND IHRER REISE AUFGEHALTEN HABEN?**		
Prä-Test: Name des Hotels		
Post-Testung: Name des Hotels		
**FRAGE 3: WELCHE PERSON(EN) HAT/HABEN SIE AUF DIESER REISE BEGLEITET? NENNEN SIE DEN/DIE VOLLSTÄNDIGEN NAMEN**		
Prä-Test: Reisegefährte/-in		
Post-Testung: Reisegefährte/-in		
**FRAGE 4: WAS WAR DER HAUPTGRUND, WIESO SIE DIESE REISE UNTERNOMMEN HABEN?**		
Prä-Test: Grund für die Reise		
Post-Testung: Grund für die Reise		
**FRAGE 5: WAS HAT IHNEN AN DIESER REISE AM BESTEN GEFALLEN?**		

Prä-Test: Gefallen an der Reise		
Post-Testung: Gefallen an der Reise		
	**Prä-Test: Summe**	
	**Post-Test: Summe**	

Patient (Name)	ID	Geburtsjahr

FRAGEBOGEN ZUM AUTOBIOGRAFISCHEN GEDÄCHTNIS – DEUTSCHE KURZVERSION DES AUTOBIOGRAFISCHEN GEDÄCHTNIS INTERVIEW (D-AMI)

TEIL 3 – SILVESTER

Prä-Test<br>Datum: __/__/___	Post-Testung<br>Datum: __/__/___	
[Interviewer: Wenn der Patient/ die Patientin sich bei der POST-Testung an ein anderes Jahr erinnert, erinnern Sie ihn/ sie bitte an das ursprüngliche Jahr und fahren mit Frage 1-5 fort. Betonen Sie, dass Sie ihn/ sie über das letzte Silvester vor Studieneintritt befragen.]		
	**Notizen**	**Summe**
ICH WERDE IHNEN EINIGE FRAGEN ÜBER LETZTES SILVESTER STELLEN: IN WELCHEM JAHR WAR DAS LETZTE SILVESTER, BEVOR SIE MIT DIESER STUDIE BEGONNEN HABEN (D.H. WELCHES JAHR WAR AM 31. DEZEMBER)?		
Prä-Test: Jahr		
**FRAGE 1: WELCHE PERSON(EN) HAT/HABEN DIESEN ABEND MIT IHNEN VERBRACHT? NENNEN SIE DEN/DIE VOLLSTÄNDIGEN NAMEN.**		
Prä-Test: Freund/-in		
Post-Testung: Freund/-in		
**FRAGE 2: WO HABEN SIE AN DIESEM ABEND GEGESSEN?**		
Prä-Test: Ort des Abendessens		
Post-Testung: Ort des Abendessens		
**FRAGE 3: WOHIN SIND SIE AN DIESEM ABEND GEGANGEN?**		
Prä-Test: Ort		
Post-Testung: Ort		
**FRAGE 4: WAS HABEN SIE DORT GEMACHT?**		
Prä-Test: Aktivität		
Post-Testung: Aktivität		
**FRAGE 5: WAS HABEN SIE UM MITTERNACHT GEMACHT?**		
Prä-Test: Mitternacht		
Post-Testung: Mitternacht		
	**Prä-Test: Summe**	
	**Post-Test: Summe**	

**Patient (Name)**	**ID**	**Geburtsjahr**

FRAGEBOGEN ZUM AUTOBIOGRAFISCHEN GEDÄCHTNIS – DEUTSCHE KURZVERSION DES AUTOBIOGRAFISCHEN GEDÄCHTNIS INTERVIEW (D-AMI)

TEIL 4 – GEBURTSTAG

Prä-Test<br>Datum: __/__/___	Post-Testung<br>Datum: __/__/___	
[Interviewer: Wenn der Patient/ die Patientin sich beim POST-Test an ein anderes Alter, als beim Prä-Test erinnert, erinnern Sie den Patienten/ die Patientin bitte an das ursprüngliche Alter und fahren mit Frage 1-5 fort.]		
	**Notizen**	**Summe**
**ICH WERDE IHNEN EINIGE FRAGEN ÜBER IHREN GEBURTSTAG STELLEN:<br>WIE LAUTET IHR GEBURTSDATUM (TAG/MONAT/JAHR)?**		
Prä-Test: Name		
[Wenn beim POST-Test ein anderes Alter genannt wird, als bei dem Prä-Test, erinnern Sie den Patienten/ die Patientin, bitte an das ursprünglich genannte Alter und fahren mit Frage 1-5 fort. Betonen Sie, dass Sie den letzten Geburtstag vor Studieneintritt erfragen. Dies muss nicht unbedingt der letzte Geburtstag sein.]		
Post-Test: Datum		
**WIE ALT WURDEN SIE BEVOR SIE MIT DIESER STUDIE BEGONNEN HABEN?**		
Prä-Test (nur): Alter		
**FRAGE 1: WELCHE PERSON(EN) HAT/HABEN MIT IHNEN IHREN LETZTEN GEBURTSTAG GEFEIERT? NENNEN SIE DEN/DIE VOLLSTÄNDIGEN NAMEN.**		
Prä-Test: Freund/-in		
Post-Testung: Freund/-in		
**FRAGE 2: WO HABEN SIE IHREN GEBURTSTAG GEFEIERT?**		
Prä-Test: Ort des Abendessens		
Post-Testung: Ort des Abendessens		
**FRAGE 3: WAS HABEN SIE DORT GEMACHT?**		
Prä-Test: Aktivität		
Post-Testung: Aktivität		
**FRAGE 4: VON WEM HABEN SIE GESCHENKE ERHALTEN (FALLS ES KEINE GESCHENKE GAB, NACH KARTEN FRAGEN)?**		
Prä-Test: Geschenk erhalten von		
Post-Testung: Geschenk erhalten von		
**FRAGE 5: WAS HABEN SIE GESCHENKT BEKOMMEN?**		
Prä-Test: Geschenk		

Post-Testung: Geschenk		
	**Prä-Test: Summe**	
	**Post-Test: Summe**	

Patient (Name)	ID	Geburtsjahr

FRAGEBOGEN ZUM AUTOBIOGRAFISCHEN GEDÄCHTNIS – DEUTSCHE KURZVERSION DES AUTOBIOGRAFISCHEN GEDÄCHTNIS INTERVIEW (D-AMI)

TEIL 5 – ARBEIT

Prä-Test<br>Datum: __/__/___	Post-Testung<br>Datum: __/__/___	
[Interviewer: Sie beginnen diesen Abschnitt, in dem sie den Patienten/ die Patientin zu seinem/ ihrem LETZTEN Job, bevor er/ sie in die Studie eingeschlossen wurde, befragen. Wenn der Patient/ die Patientin noch nie offiziell angestellt war, fragen Sie nach Ehrenämtern oder nach dem Job des Partners/ der Partnerin. Sollte der Patient/ die Patientin selbstständig sein, bitten Sie ihn/ sie, sich an den letzten Job vor der Selbstständigkeit zu erinnern.]		
[Sollte der Patient/ die Patientin sich beim POST-Test an einen anderen Job erinnern, verweisen Sie auf den ursprünglich genannten Job und das Datum, und dann fahren Sie mit Frage 1-5 fort.]		
	**Notizen**	**Summe**
**ICH WERDE IHNEN EINIGE FRAGEN ÜBER IHREN LETZTEN ARBEITSPLATZ STELLEN, AN DEM SIE TÄTIG WAREN, BEVOR SIE MIT DIESER STUDIE BEGONNEN HABEN: WIE HEISST DIE FIRMA ODER ORGANISATION, FÜR DIE SIE GEARBEITET HABEN?**		
Prä-Test: Name der Firma		
[Interviewer: Sollte der Patient/ die Patientin sich beim POST-Test an einen anderen Job erinnern, verweisen Sie auf den ursprünglich genannten Job und das Datum, und dann fahren Sie mit Frage 1-5 fort.]		
Post-Test: Name der Firma		
**ZU WELCHEM ZEITPUNKT (MONAT UND JAHR) HABEN SIE ZULETZT FÜR DIESEN ARBEITGEBER GEARBEITET, BEVOR SIE MIT DIESER STUDIE BEGONNEN HABEN?**		
Prä-Test (nur): Monat & Jahr		
**FRAGE 1: WELCHE TITEL HATTEN SIE INNE, ALS SIE ZULETZT IN DIESER ARBEITSSTELLE GEARBEITET HABEN?**		
Prä-Test: Berufsbezeichnung		
Post-Testung: Berufsbezeichnung		
**FRAGE 2: WIE HIESS IHR VORGESETZTER MIT VOR- UND NACHNAMEN?**		
Prä-Test: Name des/der Vorgesetzten		
Post-Testung: Name des/der Vorgesetzten		
**FRAGE 3: WIE LAUTETE DIE VOLLSTÄNDIGE ADRESSE DER FIRMA ODER DES UNTERNEHMENS?**		
Prä-Test: Adresse der Firma		
Post-Testung: Adresse der Firma		
**FRAGE 4: WIE LAUTETE IHRE TELEFONNUMMER, EINSCHLIESSLICH VORWAHL BEI DIESER ARBEITSSTELLE?**		

Prä-Test: Telefonnummer		
Post-Test: Telefonnummer		
**FRAGE 5: WANN (MONAT UND JAHR) HABEN SIE ANGEFANGEN, FÜR DIESEN ARBEITGEBER ZU ARBEITEN?**		
Prä-Test: Monat & Jahr		
Post-Test: Monat & Jahr		
	**Prä-Test: Summe**	
	**Post-Test: Summe**	

Patient (Name)	ID	Geburtsjahr

FRAGEBOGEN ZUM AUTOBIOGRAFISCHEN GEDÄCHTNIS – DEUTSCHE KURZVERSION DES AUTOBIOGRAFISCHEN GEDÄCHTNIS INTERVIEW (D-AMI)

TEIL 6 – ORGANISCHE BESCHWERDEN

Prä-Test<br>Datum: __/__/___	Post-Testung<br>Datum: __/__/___	
Interviewer: Sie beginnen diesen Abschnitt, in dem sie den Patienten/ die Patientin zu seinen/ ihren LETZTEN körperlichen Beschwerden befragen, wegen welcher er/ sie bei einem Arzt/-in gewesen ist, bevor er/ sie in die Studie eingeschlossen wurde. Wenn der letzte Arztbesuch des Patienten/ der Patientin eine Routine Check-up Untersuchung war, bitten Sie ihn/ sie, sich an den letzten Arztbesuch wegen körperlicher Beschwerden zu erinnern.]		
[Sollte der Patient/ die Patientin sich beim POST-Test an andere Beschwerden erinnern, verweisen Sie auf die ursprünglich genannten Beschwerden und das Datum, und dann fahren Sie mit Frage 1-5 fort.]		
	**Notizen**	**Summe**
**ICH WERDE IHNEN EINIGE FRAGEN ZU DEM LETZTEN ARZT/ DER LETZTEN ÄRZTIN STELLEN, DEN/ DIE SIE WEGEN KÖRPERLICHER BESCHWERDEN ODER EINER ERKRANKUNG AUFGESUCHT HABEN, BEVOR SIE MIT DIESER STUDIE BEGONNEN HABEN: WIESO SIND SIE ZUM ARZT GEGANGEN, WELCHE BESCHWERDEN HATTEN SIE?**		
Prä-Test: Beschwerden		
[Sollte der Patient/ die Patientin sich beim POST-Test an andere Beschwerden erinnern, verweisen Sie auf die ursprünglich genannten Beschwerden und das Datum, und dann fahren Sie mit Frage 1-5 fort.]		
Post-Test: Beschwerden		
**IN WELCHEM JAHR UND WELCHEM MONAT HATTEN SIE IHREN ARZTTERMIN?**		
Prä-Test (nur): Monat & Jahr		
**FRAGE 1: WIE HIESS DER ARZT/ DIE ÄRZTIN, DEN/ DIE SIE AUFSUCHTEN, MIT VOR- UND NACHNAMEN?**		
Prä-Test: Name des Arztes/ der Ärztin		
Post-Testung: Name des Arztes/ der Ärztin		
**FRAGE 2: WIE HIESS DAS PRAXISGEBÄUDE BZW. KRANKENHAUS UND WIE LAUTETE SEINE VOLLSTÄNDIGE ADRESSE?**		
Prä-Test: Adresse		
Post-Testung: Adresse		
**FRAGE 3: IN WELCHEM STOCK DIESES GEBÄUDES BZW. KRANKENHAUSES FAND IHR TERMIN STATT?**		
Prä-Test: Stockwerk		
Post-Testung: Stockwerk		
**FRAGE 4: WELCHE BEHANDLUNG BZW. WELCHE MEDIKAMENTE WURDE(N) IHNEN VERSCHRIEBEN?**		

Prä-Test: Medikament		
Post-Testung: Medikament		
**FRAGE 5: IN WELCHEM JAHR UND WELCHEM MONAT HABEN SIE IHRE SYMPTOME ZUERST BEMERKT?**		
Prä-Test: Monat & Jahr der Symptome		
Post-Testung: Monat & Jahr der Symptome		
	**Prä-Test: Summe**	
	**Post-Test: Summe**	

Patient (Name)	ID	Geburtsjahr

FRAGEBOGEN ZUM AUTOBIOGRAFISCHEN GEDÄCHTNIS – DEUTSCHE KURZVERSION DES AUTOBIOGRAFISCHEN GEDÄCHTNIS INTERVIEW (D-AMI)

ENDERGEBNIS

	Prä-Test Gesamtsumme	Post-Test Gesamtsumme
**Teil 1 – Familienmitglied**		
**Teil 2 – Reise**		
**Teil 3 – Silvester**		
**Teil 4 – Geburtstag**		
**Teil 5 – Arbeit**		
**Teil 6 – Organische Beschwerden**		
**Gesamtsumme**		

Part B. D-AMI Administration and Scoring Instructions (German)

ANLEITUNG ZUM AUTOBIOGRAFISCHEN GEDÄCHTNIS INTERVIEW–

DEUTSCHE KURZVERSION DES AUTOBIOGRAFISCHEN GEDÄCHTNIS INTERVIEW (D-AMI)

ANLEITUNG ZUR HANDHABUNG UND BEWERTUNG

Einführung

Dieses Dokument beschreibt die Handhabung und Bewertung des D-AMI (Kurzform). Der D-AMI wurde entwickelt um die, auf die rTMS folgende, Gedächtnisstörung zu untersuchen.

Die Kurzform des AMI (AMI-SF) wurde als eine Teilmenge des größer angelegten AMI entwickelt, welcher wiederrum auf einem ursprünglichen von Richard D. Weiner und Kollegen an der Duke Universität (Weiner et al., 1986) genutzten Instrument, basiert.

Paper von McElhiney et al. (1995), Sobin et al. (1995) und Sackheim et al. (1993, 2000) veranschaulichen die Anwendbarkeit des AMI in der EKT-Forschung.

Das primäre Ziel des AMI (Kurzform) ist es das Ausmaß der retrograden Amnesie nach einem rTMS-Intervall zu messen. Um dies zu untersuchen werden dem Patienten/ der Patientin eine Woche vor der rTMS eine Reihe von detaillierten autobiografischen Fragen gestellt. Die Fragen befassen sich mit sechs Ereignissen oder Erfahrungen, welche größtenteils im vergangenen Jahr passierten. Die gleichen Fragen werden nach Beendigung der Behandlung (POST) erneut gestellt.

Die Fähigkeit des Patienten/ der Patientin sich an spezifische Details zu erinnern, welche er/ sie ursprünglich vor der Behandlung (EKT) in dem Interview preisgegeben hatte, wird hierbei gemessen.

Auf diese Weise werden die Antworten aus dem PRÄ-Test genutzt, um als Vorlage/Vergleich in der Befragung nach der Behandlung zu dienen.

Der Vergleich der Antworten im POST und PRÄ-Test ergibt einen „Amnesie-Score“ als Prozentwert.

Da keine individuellen Normierungen entwickelt wurden, dient der „Amnesie-Score“ lediglich Forschungszwecken und nicht als klinischer Indikator.

Generelle Anweisung

Es ist wichtig vor und während der Befragung am Aufbau einer positiven Beziehung mit dem Patienten/ der Patientin zu arbeiten und dadurch eine angenehme Umgebung für den Patienten/ die Patientin zu schaffen, in der er/ sie sich sicher fühlt, die persönlichen Fragen zu beantworten und somit auch seine/ ihre Erinnerungsfähigkeiten optimal nutzt.

Der AMI (Kurzform) wird als Fragebogen vorgestellt, welcher die Fähigkeit untersucht, sich an persönliche Details aus dem eigenen Leben zu erinnern.

Dem Patienten/ der Patientin wird angekündigt, dass ein ähnlicher Test nach Beendigung der Behandlung mit rTMS durchgeführt wird. Die POST-Befragung sollte dabei nicht betont werden, da manche Patienten/ Patientinnen ansonsten, absichtlich nur bestimmte Details in der PRÄ-Befragung nennen, um in der POST-Befragung besser abzuschneiden.

Das Ziel des Fragebogens ist es, die Fähigkeit des Patienten/ der Patientin sich an vergangene Ereignisse und Erfahrungen zu erinnern, zu messen. Daher ist es wichtig einen Fokus auf die tatsächliche Erinnerung zu legen und nicht, auf die Erinnerung der PRÄ-Test Antwort.

Die Durchführungszeit ist abhängig vom klinischen und kognitiven Status des Patienten/ der Patientin, aber beträgt im Durchschnitt circa 30 Minuten. Manche Patienten/ Patientinnen sind sehr gesprächig und ausschweifend und werden gerne zusätzliche Details erzählen. Deshalb besteht eine der Aufgaben des Interviewers/ der Interviewerin darin, den Fokus zu setzten und den Patienten/ die Patientin zurück zur eigentlichen Fragestellung zu führen. Bei den sehr spezifischen Fragen zum letzten Arbeitgeber oder zur Telefonnummer/Adresse des Angehörigen können ein Teil der Patienten/ Patientinnen besorgt oder misstrauisch werden. Falls nötig, sollte der Untersucher/ die Untersucherin dem Patienten/ der Patientin erneut den Sinn des Fragebogens erklären und ihm/ ihr auch versichern, dass die genannten Personen nicht kontaktiert werden. Manche Patienten/ Patientinnen finden es zu dem beruhigend, zu wissen, dass allen Patienten/ Patientinnen die gleichen Fragen gestellt werden.

Der AMI (Kurzform) ist ein strukturierter Fragebogen und bedarf einiger klinischer Fähigkeiten, da es manchen Patienten/ Patientinnen schwerfällt diesen zu beantworten.

Der Interviewer/ die Interviewerin muss beurteilen wie viel Zeit er/ sie dem Patienten/ der Patientin pro Frage gibt. Dem Patienten/ der Patientin sollte genug Zeit gegeben werden, um sich zu erinnern, aber auch nicht so viel Zeit, dass er/ sie frustriert wird, beim Ausbleiben der richtigen Antwort. Im Verlauf kann es von Nöten sein den Patienten/ die Patientin an verschiedenen Punkten zu ermutigen, etwa mit Kommentaren wie: „Sie machen das gut.“. Dem Interviewer/ der Interviewerin sollte bewusst sein, dass es frustrierend ist, wenn man unfähig ist, sich an etwas zu erinnern und er/ sie sollte daher die Versuche des Patienten/ der Patientin wertschätzen.

PRÄ-Test Anleitung

Der D-AMI (Kurzform) hat drei Spalten für die Dokumentation der Patientenantwort (PRÄ- und POST-Test Antwort und Notizen). Der Fragebogen ist chronologisch sortiert.

Der Interviewer sollte seinen/ihren Namen und das aktuelle Datum vor dem Interview aufschreiben.

Es ist wichtig das Datum vor jedem Interview gemeinsam mit dem Patienten/ der Patientin zu besprechen, da viele Fragen das aktuelle Datum als Referenzpunkt nutzen.

Die Bewertung des AMI hängt von den im PRÄ-Test erhaltenen Antworten ab. Daher ist es essenziell, dass die Antworten so genau und vollständig wie möglich dokumentiert sind.

Je detaillierter und akkurater die Antworten des PRÄ-Tests dokumentiert worden sind, desto aussagekräftiger ist der Fragebogen als Instrument, um eine Gedächtnisstörung aufzudecken.

Alle Antworten sollten dokumentiert werden, auch Antworten wie „Ich kann es nicht sagen“ oder „Ich weiß es nicht“. Das Dokumentieren solcher Antworten ist wegweisender als eine Lücke für das zukünftige Interview, insbesondere für die Bewertung.

Nach einer kurzen Einführungsphase, welche dem Beziehungs- und Vertrauensaufbau dienen soll, sollte der Untersucher den AMI vorstellen mit folgenden Worten:

Während diesem Interview, werde ich Sie darum bitten, sich an verschiedene Erlebnisse und Erfahrungen aus Ihrer Vergangenheit, zu erinnern. Ich werde Sie darum bitten, sich an sehr spezifische Details zu erinnern. Das Interview gibt uns die Möglichkeit ihre Fähigkeit sich an Details aus Ihrer Vergangenheit zu erinnern, einzuschätzen. Ähnliche Interviews kommen im weiteren Verlauf der Studie auf Sie zu.“

PRÄ-Test Anleitung – Teil 1: Familien Mitglied

Dieser Teil des Fragebogens beginnt mit einer Einleitungsfrage nach dem wichtigsten Angehörigen des Patienten/ der Patientin, welcher nicht mit ihm/ ihr zusammenlebt.

Die zweite Frage erfragt die Beziehung des Patienten/ der Patientin zum genannten Angehörigen. Diese Fragen werden nicht bewertet, dienen aber dazu einen Angehörigen heraus zu suchen, zu dem recht genauen Fragen gestellt werden. Manche Patienten/ Patientinnen können Schwierigkeiten damit haben nur einen Angehörigen oder „den wichtigsten“ Angehörigen zu nennen. In beiden Fällen sollte der Patient/ die Patientin dazu aufgefordert werden, den Angehörigen zu nennen mit dem er/ sie den meisten Kontakt hat und über den er/ sie einige Fragen beantworten kann. Manche Patienten/ Patientinnen könnten keinen Kontakt zu Angehörigen haben, während andere keine lebenden Angehörigen mehr haben. In diesen Fällen sollen die Patienten/ Patientinnen ihren engste/-n Freund/-in nennen.

Jede Abweichung bei der Beantwortung dieser Fragen sollte in den Notizen dokumentiert werden. Die fünf folgenden Fragen erfragen Informationen über den genannten Angehörigen.

Frage 1 fragt nach dem Monat und Tag des Geburtstags der genannten Person. Die Antwort sollte dokumentiert werden, auch wenn sie nicht vollständig ist (z.B. nur Monat). Wenn der Patient/ die Patientin sagt, dass er/ sie den Geburtstag nicht weiß, sollte er/ sie zum Raten ermutigt werden. Verweigert der Patient/ die Patientin eine Antwort, dokumentieren Sie bitte „Ich weiß es nicht“ und fahren mit der nächsten Frage fort.

Frage 2 fragt nach dem Alter des Angehörigen zum Zeitpunkt des Interviews.

Diese Frage wird häufig mit einer ungefähren Schätzung, wie „Mitte Sechzig“ beantwortet. Ermutigen Sie den Patienten/ die Patientin dazu eine genauere Antwort zu geben in dem Sie fragen „Können Sie bitte genauer sein?“. Dokumentieren Sie die Antwort.

Frage 3 und 4 fragen nach der kompletten Adresse und Telefonnummer des genannten Angehörigen. Auch hier soll der Patient/ die Patientin dazu ermutigt werden, so genaue Informationen wie möglich zu geben. Sollte der Patient/ die Patientin zum Beispiel nicht spontan in der Lage dazu sein, die genaue Hausnummer oder Postleihzahl anzugeben, fordern Sie ihn dazu auf und dokumentieren Sie die Antwort. Viele Patienten/ Patientinnen werden diese Informationen nicht auswendig wissen, aber anbieten es im Adressbuch etc. nach zu schauen. Erinnern Sie den Patienten/ die Patientin daran, dass dies ein Gedächtnistest ist und jede Erinnerung, sei sie auch unvollständig, ist nützlich.

Frage 5 fragt nach der Person/ den Personen, die mit dem Angehörigen zusammen leben zu der Zeit des Interviews. Sollte der Patient/ die Patientin angeben, dass die Person alleine lebt, dokumentieren Sie diese Antwort, da im POST-Test nach der gleichen Antwort gesucht wird. Sollte die genannte Person in einer Gruppeneinrichtung, wie einem Wohnheim oder einer Anstalt leben, fragen Sie, ob sich der Patient an Namen der Mitbewohner erinnert und dokumentieren Sie diese Antwort.

PRÄ Test Anleitung – Teil 2: Reise

Dieser Abschnitt des Fragebogens beginnt mit der Frage nach dem letzten Übernachtungsausflug, vor dem Interview, welcher weiter von zu Hause weg war als 100 Meilen. Wenn der Patient/ die Patientin nicht weiter als 100 Meilen gereist ist, fragen Sie nach dem letzten Übernachtungsausflug. Wenn der Patient keinen Übernachtungsausflug unternommen hat, fragen Sie nach dem letzten Tagesausflug und ignorieren Sie Frage 2 (welche die Unterkunft abfragt). Manche Patienten erinnern sich an viele Übernachtungsausflüge, und sollten daher dazu angeleitet werden, sich an den letzten zu erinnern.

Ermutigen Sie den Patienten/ die Patientin dazu, so genau wie möglich zu antworten und stellen Sie die Dokumentation sicher. Zum Beispiel ist eine Antwort wie „Florida" im PRÄ-Test nicht so genau und daher nicht so diagnostisch bedeutend wie „Miami, Florida".

Im POST-Test wird der Interviewer/ die Interviewerin den Patienten/ die Patientin dazu anleiten, sich an den gleichen Ausflug, wie im PRÄ-Test, zu erinnern. Eine genaue und klar dokumentierte Antwort wird dies erleichtern. Im Beispiel von oben hat der Patient/ die Patientin vielleicht schon viele Reisen nach Florida unternommen, aber nur eine nach Miami.

Die zweite Frage erfragt das Datum der letzten Reise. Diese Information wird in der POST-Testung vom Interviewer/ von der Interviewerin genutzt, um die Fragen auf die gleiche Reise wie im PRÄ-Test, zu lenken.

Frage 1 fragt den Patienten/ die Patientin nach der Anzahl der Tage, inklusive Reisetage, die er/sie weg war. Diese Frage ruft oft vage Angaben wie „eine Woche oder so" hervor. Der Interviewer/ die Interviewerin sollte daher zu einer genaueren Antwort ermutigen durch die Frage „Können Sie genauer sein?". Auch sollte er es vermeiden dem Patienten/der Patientin Antworten vorzuschlagen, um die genaue Zeit zu klären. Stattdessen sollte die genauste Antwort des Patienten/ der Patientin notiert werden.

Frage 2 fragt den Patienten/ die Patientin nach dem genauen Namen des Hotels (oder der Person), wo er/sie den größten Teil der Reise verbracht hat. Der Patient/ Die Patientin könnte eine Aufforderung brauchen, um den genauen Namen anzugeben (zB „Hilton Pink Flamingo" statt nur „im Hilton" oder bei Personen „Martha Stewart" anstelle von „Martha"). Sollte der Patient/ die Patientin berichten jede Nacht an einem anderen Ort übernachtet zu haben, dokumentieren Sie die *erste Nacht*. Markieren Sie bei der Frage, dass es sich hierbei um die erste Nacht der Reise handelt, zur Orientierung beim POST-Test.

Frage 3 fragt den Patienten/ die Patienten nach der/ die Person(en), die ihn/sie auf der Reise begleitet haben. Der Patient/ die Patientin sollte dazu ermutigt werden einen Namen anzugeben, auch wenn diese Person ihn/ sie nur für einen Teil der Reise begleitet hat. Wenn der Patient/ die Patientin angibt komplett alleine gereist zu sein, sollte dies dokumentiert sein und als Erinnerung gezählt werden.

Frage 4 fragt den Patienten/ die Patientin nach dem Hauptgrund für die Reise. Er/Sie sollte ermutigt werden einen genauen Grund anzugeben, sofern dieser existiert.

Antworten mit mehreren Gründen sollten wortwörtlich dokumentiert werden (z.B. „Die Hochzeit meiner Nichte Diana und zum Urlaub machen").

Frage 5 fragt den Patienten/ die Patientin, was er/ sie am meisten an der Reise genossen hat. Diese Frage kann eine vage oder sehr allgemeine Antwort hervorrufen, daher sollte er/ sie dazu angeleitet werden noch einmal genauer nachzudenken, ob es etwas gab, dass er/ sie besonders genossen hat. Wenn der Patient/ die Patientin darauf beharrt, dass er/ sie nichts genossen hat, dokumentieren Sie das und fahren mit dem nächsten Teil des Fragebogens fort.

PRÄ-Test Anleitung – Teil 3: Silvester

Die Einleitungsfrage dieses Teils fragt den Patienten/ die Patientin nach seinem/ ihrem letzten Silvester vorm Interview.

Wie auf dem Fragebogen beschrieben sollte es dabei um den 31. Dezember gehen und nicht um den Neujahrstag. Die darauffolgenden Fragen sind von einer genauen Datierung des Jahres abhängig.

Frage 1 bittet den Patienten/ die Patientin den ganzen Namen der Person/-en zu nennen mit der/ denen er/ sie an jenem Abend Kontakt hatte. Die Frage geht direkt weiter, ob der Patient/ die Patientin eine Begleitung an den Abend hatte. Sollte der Patient/ die Patientin alleine zu einer Party gegangen sein, so soll er/ sie sagen, mit wem er/ sie auf der Party Zeit verbracht hat. Wenn der Patient/ die Patientin sich daran erinnert den größten Teil des Abends alleine gewesen zu sein, aber kurz mit einem Nachbarn gesprochen oder mit jemandem telefoniert hat, so sollte dies dokumentiert werden.

Frage 2 bittet den Patienten/ die Patientin sich daran zu erinnern wo er/ sie zu Abend gegessen hat. Für manche Patienten wird die Antwort einfach „zu Hause“ sein, während andere in einem Restaurant oder auf einer Dinnerparty gewesen sind. Der Patient/ die Patientin sollte dazu ermutigt werden sich an den Namen des Restaurants oder an den Namen der Gastgeber zu erinnern.

Frage 3 bitte den Patienten/ die Patientin sich daran zu erinnern, wohin er/ sie in dieser Nacht gegangen ist. Der Name des Clubs, der Bar, des Restaurants oder wie oben genannt der Name der Gastgeber sollten dokumentiert sein. Wenn die Antwort, der Antwort aus Frage 2 gleicht, sollte dies so dokumentiert werden.

Frage 4 bittet den Patienten/ die Patientin sich daran zu erinnern was er/ sie an dem obengenannten Ort gemacht hat. Es ist wahrscheinlich, dass hierzu eine Aufforderung für eine spezifischere Antwort gebraucht wird. Zum Beispiel könnte der Patient/ die Patientin sich folgendermaßen erinnern: „Es war eine Party, wir haben uns unterhalten.“.

Der Interviewer/ die Interviewerin könnte den Patienten/ die Patientin dann dazu auffordern sich an etwas Besonderes zu erinnern, was er/ sie getan hat, wie zum Beispiel „Wir haben viel getrunken und Rumba getanzt.“.

Auch Patienten die zu Hause geblieben sind sollten dazu aufgefordert werden eine spezifische Antwort zu geben.

Frage 5 bittet den Patienten/ die Patientin dazu sich daran zu erinnern, was er/ sie an Mitternacht getan hat. Auch hier könnte eine Aufforderung von Nöten sein, um eine spezifische Antwort zu erhalten.

PRÄ-Test Anleitung – Teil 4: Geburtstag

Die Einleitungsfrage fragt den Patienten/ die Patientin nach seinem/ ihrem Geburtstag und wie alt er/ sie beim letzten Geburtstag vor dem Interview geworden ist. Es ist sinnvoll hier kur nachzurechnen, um sicher zu gehen, dass der Patient/ die Patientin das richtige Alter angibt.

Darauffolgende Interview Sitzungen werden sich auf den Geburtstag beziehen und daher ist es sinnvoll, wenn der Patient sich an den richtigen Geburtstag erinnert. Die meisten Patienten/ Patientinnen werden sich nicht an den tatsächlichen Geburtstag erinnern, sondern die größte Feierlichkeit nennen.

Frage 1 bittet den Patienten/ die Patientin die vollen Namen der Person/ -en zu nennen, welche mit ihm/ ihr an seinem/ ihrem letzten Geburtstag gefeiert haben. Wenn der Patient/ die Patientin angibt, dass niemand an seinen/ ihren Geburtstag gedacht hat oder niemand mit ihm/ ihr gefeiert hat, ist dies so zu dokumentieren.

Frage 2 bittet den Patienten/ die Patientin sich daran zu erinnern, wo er/ sie diesen Geburtstag gefeiert hat. Wenn der Patient/ die Patientin antwortet, dass er/ sie nicht gefeiert hat, sollte der Interviewer/ die Interviewerin den Patienten/ die Patientin darum bitten sich daran zu erinnern, wo er/ sie den Tag verbracht hat.

Frage 3 bittet den Patienten/ die Patientin darzulegen was er/ sie an der obengenannten Lokation getan hat. Der Patient kann ggf. Ermutigungen brauchen, um eine genaue Antwort zu geben.

Frage 4 bittet den Patienten/ die Patientin die Namen der Person/ -en aufzuzählen, von denen er/sie Geschenk erhielt. Wenn der Patient/ die Patientin keine Geschenke bekam, soll er/ sie die Namen der Personen aufzählen, von denen er/ sie Karten erhalten hat.

Es sollte eine Anmerkung (Geschenk/ Karte) hinter dem Namen jeder Person gemacht werden. Dies wird dem Interviewer/ der Interviewerin in Zukunft helfen, die Liste der Namen zu erfragen. Der Patient/ die Patientin soll die genauen Namen der Personen angeben. Eine Antwort wie „Ich habe Karten von allen meinen Kindern bekommen“, ist nicht ausreichend genug. Der Patient/ die Patientin soll dazu aufgefordert werden den ganzen Namen seiner/ ihrer Kinder zu nennen.

Frage 5 bittet den Patienten/ die Patientin sich an Geschenke zu erinnern. Bekam er/ sie keine Geschenke, sollte diese Frage ausgelassen werden. Patienten werden manchmal mit spekulativen Aussagen antworten wie „Ich bekomme für gewöhnlich jedes Jahr einen Pullover von meiner Nichte, also werde ich das wahrscheinlich bekommen haben“.

Der Patient/ die Patientin sollte gebeten werden sich genau an das Geschenk zu erinnern durch eine Frage wie: „Können Sie den Pullover beschreiben, den Sie bekommen haben?“. Die Geschenke sollten mit so vielen Details wie möglich dokumentiert werden (Bsp.: eine blaue Fliege, ein grüner Pullover). Wenn der Patient/ die Patientin Geld bekommen hat, sollte er/ sie sich an die Höhe der Summe erinnern.

PRÄ-Test Anleitung – Part 5: Anstellung

Dieser Abschnitt beginnt mit der Frage nach der letzten Firma/ Organisation für die der Patient/ die Patientin gearbeitet hat, bevor er/ sie mit der Studie begonnen hat. Wenn der Patient/ die Patientin selbstständig ist, soll er/ sie sich an den letzten Job vor der Selbstständigkeit erinnern. Wenn der Patient/ die Patientin noch nie oder seit 20 Jahren nicht mehr außer Haus gearbeitet hat, fragen Sie nach ehrenamtlichen Tätigkeiten. Sollte auch dies nie ausgeübt worden sein, fragen Sie nach der Arbeit des Partners/ der Partnerin. Bitte jegliche Abweichung an die Originalfrage dokumentieren. Die zweite Einleitungsfrage fragt den Patienten/ die Patientin nach dem Monat und Jahr, in welchem er/ sie zuletzt für den Arbeitgeber gearbeitet hat. Die Antwort wird nicht mit Punkten bewertet, aber für den POST-Test zur Erinnerung benutzt.

Frage 1 bittet den Patienten/die Patientin sich an seine/ ihre letzte Berufsbezeichnung bei der obengenannten Arbeitsstelle zu erinnern. Der Patient/ Die Patientin soll sich hier an die offizielle Bezeichnung erinnern.

Frage 2 bittet den Patienten/ die Patientin sich an den Namen des/ der Vorgesetzten zu erinnern. Wenn der Patient/ die Patientin keine/ -n Vorgesetzte/ -n hatte, fragen Sie nach seinem/ ihrem nächsten Höhergestellten und notieren Sie die Position. Der/ die nachfolgende(n) Interviewer/ -in muss wissen ob er/ sie den Patienten/ die Patientin nach einem/ einer Vorgesetzten oder nächst Höhergestellten fragen muss.

Frage 3 und 4 bitten den Patienten/ die Patientin sich an die vollständige Adresse und Telefonnummer des Gebäudes zu erinnern, in dem er/ sie gearbeitet hat. Der Patient/ die Patientin kann eine Aufforderung brauchen, um auch die Postleihzahl zu nennen. Wenn der Patient/ die Patientin keinen eigenen Telefonanschluss hatte, bitten Sie ihn/ sie darum, sich an die allgemeine Firmennummer zu erinnern.

Frage 5 bittet den Patienten/ die Patientin sich an den Monat und das Jahr zu erinnern, in welchem er/ sie angefangen hat für den Arbeitgeber zu arbeiten. Ermutigen Sie den Patienten/ die Patientin so genau wie möglich zu antworten und dokumentieren Sie die Antwort, auch wenn es nur das Jahr ist.

PRÄ-Test Anleitung – Teil 6: körperliches Leiden

Die erste Einleitungsfrage bittet den Patienten/ die Patientin sich an den letzten Arztbesuch zu erinnern, den er/ sie wegen körperlicher Beschwerden oder Krankheit unternommen hatte.

Der Patient/ die Patientin wird gefragt wieso er/ sie zum Arzt gegangen ist, in dem er/ sie die Beschwerden benennt. Sollte der letzte Arztbesuch ein Check-up gewesen sein, bitten Sie den Patienten/ die Patientin darum sich an das letzte Mal zu erinnern, bei dem er/ sie wegen organischer Beschwerden beim Arzt gegangen war. Notieren Sie dies im vorhergesehenen Kästchen.

Des Öfteren werden Patienten die Besuche bei ihrem Psychiater aufzählen, da dies der Arzt ist, den sie am häufigsten aufsuchen und da manche der psychiatrischen Erkrankungen auch somatische Symptome beinhalten. Die Frage sollte umformuliert werden und die Betonung sollte dieses Mal auf den *körperlichen* Beschwerden liegen.

Die zweite Einleitungsfrage fragt nach dem Monat und Jahr des Besuchs. Wie alle vorangegangen Einleitungsfragen, wird dies nicht bewertet, kann aber eine nützliche Hilfe sein, falls der Patient/ die Patientin sich in der folgenden Fragesitzung nicht mehr an den Besuch erinnert.

Frage 1 bittet den Patienten/ die Patientin sich an den Vor- und Nachnamen des Arztes/ der Ärztin zu erinnern, den er/ sie als letztes aufgesucht hat. Sollte der Patient/ die Patientin vorerst nur den Nachnamen sagen, fordern Sie ihn/ sie auf, sich auch an den Vornamen zu erinnern.

Frage 2 bittet den Patienten/ die Patientin sich an die Adresse des Gebäudes/ Krankenhauses, wo er/ sie den og. Arzt aufgesucht hat, zu erinnern. Der Patient/ die Patientin kann sich ggf. nicht an die komplette Adresse erinnern, notieren Sie was erinnert wird.

Frage 3 bittet den Patienten/ die Patientin sich an das Stockwerk auf welchem der Termin stattfand zu erinnern. Dies kann eine Wiederholung sein, falls das Stockwerk schon in der vorhergehenden Frage genannt wurde. Nichts desto trotz sollte das Stockwerk im entsprechenden Kästchen dokumentiert sein.

Frage 4 bittet den Patienten/ die Patientin sich an die Behandlung oder die Medikamente, welche während des Arztbesuchs verschrieben worden sind, zu erinnern. Alle Verschreibungen sollten notiert werden, auch wenn es sich dabei um Rezeptverlängerungen gehandelt hat. Dosierung und Einnahmeplan müssen nicht notiert werden. Vorschläge des Arztes wie „Bettruhe“ sollten dokumentiert und bewertet werden.

Frage 5 bittet den Patienten/ die Patientin sich an den Monat und das Jahr zu erinnern, in welchem er die Symptome zuerst bemerkte. In manchen Fällen kann der Erkrankungsbeginn vor vielen Jahren gewesen sein oder seit Geburt bestehen. Der Patient/ die Patientin sollte dazu ermutigt werden so genau wie möglich zu antworten.

POST-Testung Anleitung

Der eigentliche POST-Test sollte im Zeitraum von 24 – 72 Stunden nach der letzten EKT-Behandlung durchgeführt werden. Es wird empfohlen, dass die Person, welche den PRÄ-Test mit dem Patienten/ der Patientin durchgeführt auch den POST-Test durchführt. Das Interview wird angenehmer verlaufen, wenn es von einem Interviewer/ einer Interviewerin durchgeführt wird, der/die den Patienten/ die Patientin und die Antworten aus dem PRÄ-Test bereits kennt.

Sollte dies nicht möglich sein, sollte der neue Interviewer/ die neue Interviewerin sich davor ein paar Minuten Zeit nehmen, um die Antworten des PRÄ-Tests durch zu lesen und sich ggf. Notizen von Abweichungen machen (z.B.: der Patient ist selbstständig und alle Fragen bezüglich der Arbeitsstelle beziehen sich auf die Zeit vor der Selbstständigkeit).

Zu Beginn des Tests sollte der Interviewer/ die Interviewerin das Datum und seinen/ ihren Namen auf dem Fragebogen dokumentieren. Der Interviewer/ die Interviewerin sollte die Aufgaben einläuten in dem er folgendes Statement gibt:

Während diesem Interview, werde ich Sie darum bitten, sich an verschiedene Erlebnisse und Erfahrungen aus Ihrer Vergangenheit, zu erinnern. Ich werde Sie darum bitten, sich an sehr spezifische Details zu erinnern. Das Interview gibt uns die Möglichkeit ihre Fähigkeit sich an Details aus Ihrer Vergangenheit zu erinnern, einzuschätzen.

Der Patient/ die Patientin erinnert sich womöglich nicht an die vorhergehende Fragesitzung.

Manche Patienten werden versuchen sich an die Antwort des vorhergehenden Tests zu erinnern und sich darauf konzentrieren wie gut sie im Vergleich dazu abschneiden.

Diese Patienten sollten dazu ermutigt werden sich auf die aktuellen Fragen zu konzentrieren und sich verbunden damit, auch an die tatsächlichen Erinnerungen erinnern. Manchmal können ein paar aufmunternde Worte sehr viel dazu beitragen, dass der Patient/ die Patientin sich entspannt und sich der eigentlichen Aufgabe widmet. Manche Patienten, die sich nicht sofort an das vorhergehende Interview erinnern, können agitiert oder nervös werden, wenn sie bemerken, dass sie diese Fragen schon in einem vorhergehenden Setting gefragt worden sind. Dem Patienten/ der Patientin sollte erklärt werden, dass er/ sie ähnliche Fragen bereits gestellt bekommen hat und dass es nicht unüblich ist für manche Menschen, dass man dies vergisst.

Nach der Einleitung der Aufgaben, fahren sie fort mit der Befragung, aber dieses Mal werden alle Fragen, die beim PRÄ-Test nicht gefragt worden sind, oder mit 0 Punkten bewertet worden sind, ausgelassen. Nochmals, das Interview versucht zu zeigen, welche Erinnerungen verloren gegangen sind und nicht welche hinzugekommen sind.

Wenn eine Frage beim PRÄ-Test nicht komplett beantwortet wurde, wie zum Beispiel der Monat des Geburtstags eines Angehörigen, aber ohne Angabe des Tages, sollte nun die gleiche Frage wie im PRÄ-Test gestellt werden ohne den Patienten dazu aufzufordern jetzt genauer zu antworten. Wenn der Patient/ die Patientin spontan eine genauere Antwort, als beim PRÄ-Test gibt, sollte dies so aufgenommen werden, auch wenn es die Bewertung nicht beeinflusst.

Bei der Anleitung der Fragen jedes Fragebogenteils, soll der Interviewer/ die Interviewerin gemeinsam mit dem Patienten/ der Patientin schauen, ob er/ sie sich an die gleiche Person oder das gleiche Event erinnert. Zum Beispiel bei der Frage nach dem nahestehenden Angehörigen aus dem ersten Teil, könnte der Patient einen anderen Namen nennen, als bei der PRÄ-Testung, wie zB „Robert“ anstelle von „Joe“.

Dieser neue Name sollte in dem dafür vorgesehenen Kästchen eingetragen werden und dann sollte der Patient/ die Patientin daran erinnert werden, dass er/ sie im ersten Interview von „Joe“ gesprochen hat, und gefragt werden, ob er/ sie sich an „Joe“ erinnert. Wenn der Patient/ die Patientin sich an diese Person erinnert, sollte dies so dokumentiert werden. Dem Patienten/ der Patientin sollte dann erklärt werden, dass nun einige Fragen zu „Joe“ gestellt werden. Es ist unwahrscheinlich, dass der Patient/ die Patientin sich nicht an den Angehörigen/ die Angehörige erinnern. Wenn der Patient sich partout nicht an die ursprüngliche Person erinnert, sollte der Interviewer/ die Interviewerin diesen Teil überspringen und mit 0 Punkten werten.

Während des POST-Tests könnte klar werden, dass der Patient/ die Patientin sich fälschlich beim ersten Interview geäußert hat. Zum Beispiel kann einem Patienten klar werden, dass die Reise, von der er/ sie beim PRÄ-Test sprach, nicht die letzte Reise vor Studieneintritt war. In diesem Fall sollte der Interviewer/ die Interviewerin darauf bestehen, dass über die erst-genannte Reise gesprochen wird und einen Vermerk über die neue Erinnerung des Patienten/ der Patientin machen.

Es ist wichtig, dass der Interviewer/ die Interviewerin prüft ob der Patient/ die Patientin alle Informationen vom PRÄ-Test wiedergeben kann. Die Antworten sollten klar und vollständig notiert werden, damit sie mit dem PRÄ-Test verglichen werden. Wie im PRÄ-Test sollten Antworten wie „Ich weiß es nicht“ oder „keine“ dokumentiert werden, da sie mehr bedeuten als eine Lücke.

Aufforderung zu einer genaueren Antwort sind beim POST-Test anders als beim PRÄ-Test. Der Patient/ die Patientin könnte mehr Hilfestellung brauchen, um sich an die Antworten aus dem PRÄ-Test zu erinnern. Zum Beispiel wenn der Patient/ die Patientin behauptet eine Reise mit „Mary“ unternommen zu haben, sollte der Interviewer/ die Interviewerin nachprüfen ob der Patient sich an Mary’s Nachnamen erinnert, *wenn* dieser im PRÄ-Test genannt wurde. Wenn der Patient/ die Patientin eine Person weglässt, mit der er gereist ist, sollte gefragt werden ob noch jemand dabei war.

Dem Patienten/ der Patientin sollte genug Zeit und Ermutigung gegeben werden, um alle Mitreisenden auszuzählen, bis zu dem Punkt an dem klar wird, dass das Erinnerungsvermögen des Patienten/ der Patientin erschöpft ist.

In der Absicht den Test besser zu verwalten und korrekt zu bewerten, wurden die Schlagworte, die den Test ausmachen zusammengefasst und in fünf Kategorien eingeteilt: Name/Ort, Datum/Zahlen, Adresse, Aufzählung und Beschreibung. Statt von Punkt zu punkt zu verfahren, werden die Hauptpunkte des POST-Tests in diesen Kategorien zusammenhängend erklärt.

Anhang 1 beinhaltet eine Tabelle, in welcher die Fragen numerisch aufgelistet sind mit der zugeschriebenen Kategorie.

POST-Test Anleitung: Name/ Ort Fragen

Die sieben Name/Ort Fragen, bitten den Patienten/ die Patientin sich an die Namen von Menschen (Bsp.: Arzt, Vorgesetzter) und Orten (Bsp.: Geburtstagsfeier, Silvester und Ziel der letzten Reise) zu erinnern. Der Interviewer/ die Interviewerin sollte die Antwort des PRÄ-Tests lesen und mit sanftem Nachdruck nachforschen ob der Patient/ die Patientin fähig dazu ist, sich an den Namen oder Ort genauso detailreich zu erinnern, wie beim PRÄ-Test. Sollte sich ein Patient/ eine Patientin beispielsweise beim PRÄ-Test an den Vor- und Nachnamen seines Arztes erinnern, und beim POST-Test nur den Nachnamen nennen, dann sollte der Interviewer/ die Interviewerin den Patienten/ die Patientin dazu auffordern den vollständigen Namen zu sagen. Eine passende Nachfrage könnte hier sein: „Können Sie sich an den Vornamen Ihres Arztes erinnern?“

Der Interviewer/ die Interviewerin sollte *nicht* sagen: „Letztes mal nannten Sie Vor- und Nachnamen des Arztes. Wie ist der Vorname?“. Unabhängig davon ob der Patient/ die Patientin einen zweiten Anlauf bei der Erinnerung des Namens braucht, sollte ein Vermerk gemacht werden wie „Pat. Erinnert sich nicht an den Namen“ oder „Der Name ist Markus“. Wenn der Patient/ die Patientin einen anderen Namen oder Ort angibt als beim PRÄ-Test, sollte der Patient/ die Patientin nochmal gefragt werden, ob es möglicherweise auch eine andere Antwort auf diese Frage geben könnte. Beispielsweise könnte sich ein Patient/ eine Patientin im PRÄ-Test daran erinnern, dass sein/ ihr Vorgesetzter „Larry Tate“ hieß. Beim POST-Test sagt er/ sie jedoch „Mr. Drisdale“. Der Interviewer/ die Interviewer sollte dann fragen, ob der Patient/ die Patientin ggf. noch andere Vorgesetze hatte und was deren Namen waren. Zudem sollte der Interviewer/ die Interviewerin sich versichern, dass es um den gleichen Job, wie beim PRÄ-Test geht.

POST-Test Anleitung: Datum/ Anzahl – Fragen

Die acht Datum/ Anzahl Fragen verlangen Daten des Patienten/ der Patientin, wie der Monat und Tag des Geburtstags eines Angehörigen und den Monat und Tag des letzten Arztbesuchs; Zahlen wie das Alter des Angehörigen, die Anzahl an Urlaubstagen und Telefonnummern. Auch hier ist das Ziel zu sehen, ob der Patient/ die Patientin das gleiche spezifische Erinnerungsvermögen hat wie beim PRÄ-Test. Wenn sich der Patient/ die Patientin zum Beispiel an eine Telefonnummer ohne Vorwahl erinnert, muss der Interviewer/ die Interviewerin nachfragen, ob er/ sie sich auch an diese noch erinnert. Wenn der Patient/ die Patientin eine Antwort gibt, welche unspezifischer oder eher vage im Vergleich zur Antwort vom PRÄ-Test ist, muss der Interviewer genauer nachfragen. Beispiel: Ein Patient erinnert sich daran, dass der Angehörige, der ihm am nächsten steht „etwas Anfang 60“ ist, hat aber beim PRÄ-Test angegeben, dass der Angehörige 63 ist. Jetzt sollte der Interviewer nachfragen: „Können Sie etwas genauer sein?“. Sollte der Patient sich nicht an eine Antwort, welche er im PRÄ-Test noch wusste, erinnern, kann es sein, dass er einen Moment Zeit braucht, bis die Erinnerung wiederkommt. Wenn jedoch klar wird, dass der Patient sich nicht mehr erinnern kann, soll mit dem Interview fortgefahren werden mit einem Angebot wie: „Das ist in Ordnung, wenn Ihnen die Antwort, doch noch einfällt im Laufe des Interviews, lassen Sie mich das wissen. Hier ist die nächste Frage.“

POST-Test Anleitung: Adressangaben – Fragen

Drei Fragen im Fragebogen D-AMI SF fordern den Patienten/ die Patientin dazu auf, sich an die Adressen des/ der Angehörigen, des Arbeitsplatzes und der Arztpraxis zu erinnern. Der Patient/ die Patientin wird ein gezieltes Nachfragen benötigen, um auch hier *alle* Anteile der Adressen zu nennen, welche er beim Prä-Test genannt hat. Die Bewertung dieser Fragen, hängt vom Vergleich der Antwort des POST-Tests mit der Antwort des PRÄ-Tests ab. Sogar Details wie die Apartmentnummer, werden als Teil der Antwort gezählt. Daher ist es wichtig, den Patienten/ die Patientin zu ermutigen, jedes Detail, an welches er/ sie sich erinnert, zu nennen. Eine angemessene Nachfrage könnte hier sein: „Gibt es eine Apartmentnummer?“ oder „Erinnern Sie sich an die Postleihzahl?“ Es ist unnötig nach Details nach zu harken, welche schon beim PRÄ-Test nicht vorhanden waren.

POST-Test Anleitung: Aufzählungsfragen

Die sechs Aufzählungsfragen erfordern Listen, wie mit wem der Patient seinen letzten Geburtstag oder Silvester feierte, mit wem er/ sie verreist war und von wem er/ sie Geschenke erhalten hat. Wie bei den anderen Fragetypen, sollte der Untersucher/ die Untersucherin auch hier mit den Antworten des PRÄ-Tests vertraut sein und den Patienten dazu auffordern eine ähnliche Antwort zu geben. Auch wenn die Antwort des PRÄ-Tests keine Liste war, sondern eine Aussage wie „Ich habe den Abend alleine verbracht“, sollte die Frage beim POST-Test gestellt werden, um sehen zu können, ob der Patient/ die Patientin sich noch an das gleiche erinnert.

POST-Test Anleitung: Beschreibende Fragen

Die sechs beschreibenden Fragen bitten den Patienten/ die Patienten darum, sich an ein Ereignis, wie den Grund für die letzte Reise, was er/ sie an der Reise genossen hat, oder was er/ sie an Mitternacht vom Silvesterabend gemacht hat, zu erinnern. Der Interviewer wird selbst beurteilen müssen, ab wann die Erinnerung des Patienten erschöpft ist. Diese Art von Fragen rufen Antworten hervor, die nicht sachlich und mehr tagesformabhängig sind. Konsequenz daraus ist, dass die Antwort anders als beim PRÄ-Test sein kann, aber immer noch korrekt ist. Beispielsweise kann ein Patient beim PRÄ-Test angegeben haben, er hätte letztes Silvester um Mitternacht „angestoßen mit Freunden und getanzt“. Beim POST-Test könnte die Antwort sein, er habe seine Frau geküsst. Obwohl die Antwort des POST genauso richtig sein kann, sollte der Interviewer/ die Interviewerin herausfinden ob sich der Patient/ die Patientin nach wie vor an die Aktivität erinnert, welche er/ sie im PRÄ-Test beschrieben hatte. Dies könnte gelingen durch eine Folgefrage wie: „An was erinnern Sie sich noch, was sie auch um Mitternacht herum getan haben?“. Der Interviewer/ die Interviewerin sollte mit dem Nachfragen fortfahren, bis er die ursprüngliche Antwort erhält oder merkt, dass das Erinnerungsvermögen des Patienten an dieser Stelle erschöpft ist.

BEWERTUNG DES D-AMI SF

Der D-AMI SF wird bewertet, indem die Antworten des POST-Tests mit den Antworten des PRÄ-Tests verglichen werden. Ebenso wie die Anleitung des PRÄ-Tests, ist auch die Bewertung anders, da es noch keine Vergleichserinnerung gibt.

Die Anleitung zur Bewertung des PRÄ-Tests wird hier zuerst aufgeführt, mit einer anschließenden Gliederung für die Bewertung des POST-Tests.

PRÄ-Test Bewertung

Bei der Bewertung des PRÄ-Tests geht es vor allem darum, herauszustellen, welche Fragen genau genug beantwortet wurden, um zu gewährleisten, dass ein Vergleich zu den POST-Test Fragen möglich ist. Zu dem ergibt der PRÄ-Test eine genaue Zahl, durch welche das Ergebnis des POST-Tests geteilt wird, um den letztendlichen Amnesie-Wert zu erfahren. Hierbei wird vorgeschlagen, dass die Person, welche die Interviews führt, auch für die Auswertung des Tests verantwortlich ist. Als Zeitpunkt für die Auswertung empfiehlt es sich dies am besten direkt nach der Befragung zu machen, damit die Begegnung mit dem Patienten noch recht gegenwärtig ist.

Die folgenden Regeln sind auf die Bewertung des PRÄ-Tests in Form von Punkten anzuwenden.

Jede im Nachhinein als **vollständig befunden Antwort** aus dem Erinnerungsvermögen, erhält **2 Punkte**.

Jede Antwort, bei welcher **keine Erinnerung** vorliegt („Ich weiß es nicht“, „Ich kann mich nicht erinnern“) erhält **0 Punkte**.

Eine im PRÄ-Test gegeben Antwort kann also nur 2 oder 0 Punkte erzielen. Es gibt keinen Zwischenwert wie 1 Punkt. Dies ist ein Unterscheid zur Bewertung des POST-Tests, welcher im Gegenzug den Wert 1 Punkt für teilweise richtige Antworten, beinhaltet.

Die Bewertung der Antworten des PRÄ-Tests ist also eher milde und großzügig. Manch unvollständige Antwort, wie unten genauer ausgeführt, wird hier mit 2 Punkten bewertet, so lange die Antwort genaue Details liefert. Nochmals, Ziel des PRÄ-Tests ist es so viel ausführliche Information wie möglich, als Grundlage für den Vergleich, zu sammeln.

Wie oben beschrieben wurden die gemeinsamen Merkmale der Fragen des D-AMI in fünf Kategorien zusammengefasst:

Name/ Ort,

Datum/ Anzahl,

Adressangaben

Aufzählungen und

Beschreibungen.

Jede dieser fünf Kategorien hat leicht unterschiedliche Bewertungsregeln und wird daher separat erläutert. Eine Tabelle mit der Fragenummer und der zugehörigen Kategorie befindet sich im Anhang 1.

PRÄ-Test Bewertung: Name/Ort Fragen

Die sieben Name/ Ort Fragen, bei welchen der Patient sich an den Namen eines Menschen oder eines Ortes erinnern muss, sind meist kurz und enthalten eine exakte Antwort. Jede Antwort, welche eine deutliche Erinnerung, wie „Wir feierten bei Tante Agatha zu Hause“ oder „Ich wurde zum Essen im 21 Club ausgeführt“ oder „Mein Arzt heißt Dr. Welby“ wird mit 2 Punkten bewertet.

Sogar Antworten, die ungenau sind wie zB „Dr. Welby“ anstelle von „Dr. Marcus Welby“ oder „Hilton Hotel“ anstelle von „Hilton Pink Flamingo Hotel“, werden mit 2 Punkten bewertet, so lange es sich hierbei um eine eindeutige Erinnerung handelt. Ein Vor- oder Nachname muss mindestens angegeben werden, oder wenn ausdrücklich nach Vor- und Nachnamen gefragt wird, muss beides vorhanden sein um 2 Punkte zu erreichen. Ähnlich hierzu muss auch mindestens der halbe Name des Restaurants oder Hotel genannt werden, um 2 Punkte zu erreichen.

Antworten wie, „Wir schliefen in einem Hotel, aber den Namen habe ich vergessen“ oder „Wir müssen Essen gewesen sein“ werden mit 0 Punkten bewertet, genau wie ein kompletter Erinnerungsverlust, wie „Ich erinnere mich nicht.“.

PRÄ-Test Bewertung: Datum/ Zahl Fragen

Die Datum-/ Anzahlfragen erfordern vom Patienten eine Angabe von Daten wie der Monat und Tag des Geburtstags eines Angehörigen und Zahlen wie, Anzahl der Reisetage des letzten Urlaubs und Telefonnummern. Jede Antwort die komplett und genau ist, wie „Wir waren für 2 Wochen weg“ oder „Er ist 62 Jahre alt“ erhält 2 Punkte. Eine Antwort, die genau ist, aber nicht vollständig, wie zum Beispiel „Sein Geburtstag ist im August, aber ich weiß nicht an welchem Tag genau“, wird auch mit 2 Punkten bewerten. Der Patient muss sich mindestens an den Monat des erinnern, wenn nach Monat und Tag gefragt wird und mindestens an das Jahr erinnern, wenn nach Jahr und Monat gefragt wird. Gleiches gilt für die Telefonnummer und Postleihzahl.

Ähnlich dazu erfordern die drei Fragen, die eine sehr genaue Nummer (Alter des Verwandten, Stockwerk des Arzttermins, Anzahl der Urlaubstage) erfragen, mindestens ein spezifisches Spektrum an Zahlen, um zwei Punkte zu erhalten. Antworten wie: „Der Termin war entweder im dritten oder vierten Stock“ und „Er ist entweder 62 oder 63 Jahre alt“ sind plausibel genug um 2 Punkte zu erhalten. Obgleich es besser von dem Interviewer/ der Interviewerin gewesen wäre, wenn der Patient dazu aufgefordert worden wäre eine spezifischere Antwort zu geben.

Jede Antwort, die zu allgemein gefasst ist wie: „Er ist über 60“ oder „Sein Geburtstag ist im Sommer“, ergibt 0 Punkte.

PRÄ-Test Bewertung: Adress-Fragen

Da viele Patienten sich nicht genau an die Apartmentnummer oder die Postleihzahl erinnern werden, erhalten Antworten, die mindestens den Namen der Straße, Stadt und Bundesland enthalten, 2 Punkte. Jede Antwort die weniger Information als das enthält, bekommt 0 Punkte.

PRÄ-Test Bewertung: Aufzählungsfragen

Wenn der Patient/ die Patientin Gegenstände oder Namen für die Auflistung nennen kann, auch wenn es nur ein Name oder ein Gegenstand ist, erhält er/ sie 2 Punkte. Wenn der Patient/ die Patientin glaubhaft versichert, dass keine Gegenstände oder Namen auf der Liste sein sollen, da er/ sie Beispielsweise alleine verreist war oder keine Geschenke/ Karten bekommen hat, ist die Punktezahl hier 2. Wenn der Patient/ die Patientin jedoch zugibt, sich nicht erinnern zu können, ist diese Antwort mit 0 Punkten zu bewerten.

PRÄ-Test Bewertung: Beschreibungen

Um die sechs Beschreibungen zu erfragen, muss der Untersucher/ die Untersucherin feststellen, ob der Patient/ die Patientin sich an eine bestimmte Erinnerung erinnert. Jede Antwort die eine klare Erinnerung wie „Wir fuhren nach Florida, um der Hochzeit meiner Nichte beiwohnen zu können“ oder „Wir küssten uns um Mitternacht“ wiedergibt, wird mit 2 Punkten bewertet. Wenn der Patient nicht sonderlich aktiv oder gesellig war, kann die Antwort zu diesen Fragen eingeschränkt sein. Beispielsweise könnte es sein, dass der Patient an Silvester früh ins Bett gegangen ist und daher um Mitternacht bereits schlief. Auch diese Antwort ist als klare Erinnerung zu werten und erhält 2 Punkte. Antworte wie „Ich erinnere mich nicht mehr“ jedoch, werden mit 0 Punkten bewertet.

Zusammenfassung der PRÄ-Test Bewertung

Nach der Auswertung des Interviews sollte jede Frage einen Punktestand von 0 oder 2 haben. Das Ergebnis von allen Fragen sollte addiert werden und unter der „Gesamtpunktzahl“ des PRÄ-Tests verbucht werden. Die „Gesamtpunktezahlen“ der einzelnen Abschnitte sollten dann übertragen werden auf die letzte Seite und anschließend noch mal zur Gesamtpunktezahl des ganzen PRÄ-Tests addiert werden. Diese Gesamtpunktezahlen werden später dann genutzt, um den „Amnesie-Score“ der verschiedenen Teile mit dem POST-Test zu ermitteln.

POST-Test Bewertung

Das Ziel der Bewertung des POST-Tests ist es, die Antworten mit denen des PRÄ-Tests zu vergleichen. Wenn die Antwort beim POST-Test **identisch** mit der Antwort beim PRÄ-Test ist, wird sie mit **2 Punkten** bewertet. Nochmals, ein **Erinnerungsverlus**t wie „Ich weiß es nicht" zählt als **0 Punkte**. Wenn die Antwort beim POST-Test **teilweise richtig** ist, also ein Teil ist richtig wiedergegeben worden, aber es fehlt ein weiterer Teil, verglichen mit dem PRÄ-Test, so wird dies mit **1 Punkt** bewertet.

Um genauer abzuschätzen, ob eine Antwort als Teilleistung gezählt werden kann, dienen die Hilfestellungen zu den fünf einzelnen Fragetypen.

POST-Test Bewertung: Name/ Ort Fragen

Jeder Name, welcher beim POST-Test genannt wird und mit der Antwort vom PRÄ-Test identisch ist, wird mit 2 Punkte bewertet. Die Antwort beim POST-Test könnte in der Abfolge abweichen von der des PRÄ-Tests. Beispielsweise: „Hilton, Pink Flamingo" oder „The Pink Flamingo Hilton". Da beide Antworten im Grunde das gleiche Detailerinnerungsvermögen abbilden, werden hierfür 2 Punkte vergeben.

Antworten dieses Fragetyps haben für gewöhnlich mehrere Bestandteile, wie zum Beispiel Vor- und Nachname. Jede Antwort des POST-Tests, bei der auch nur ein nicht essentieller Bestandteil nicht erinnert werden kann oder falsch wiedergegeben wird, wird mit nur 1 Punkt bewertet. Ein nicht essentieller Bestandteil wird definiert als Teil eines Namens des Ortes oder einer Person, ohne den der Ort oder die Person trotzdem noch identifiziert werden kann. Beispielsweise das Wort „Club" im Namen des Restaurants „21 Club" ist zur Identifikation nicht nötig. Wenn es beim POST-Test weggelassen wird, wird diese Antwort mit 1 Punkt bewertet. Ähnlich bei Antworten wie „Dr. Welby" beim POST-Test und „Dr. Marcus Welby" beim PRÄ-Test (bewertet mit 1 Punkt). Eine Ausnahme stellt der Fall dar, dass bereits im PRÄ-Test nur eine unvollständige Antwort gegeben wurde und beim POST-Test auch diese Antwort nicht wiedergegeben werden kann. Beispielsweise wurde schon beim PRÄ-Test nur mit „Dr. Welby" geantwortet und beim POST-Test dann mit „Dr Wealthy". Hier ist die korrekte Bewertung 0 Punkte.

Genauso verhält es sich mit dem Fall, dass beim PRÄ-Test schon keine Erinnerung vorhanden war. Hier ist die Bepunktung bei beiden Testdurchläufen 0 Punkte.

POST-Test Bewertung: Datum/ Zahl Fragen

Wenn die Antwort des POST-Tests alle Bestandteile hat und zu dem identisch mit der Antwort des PRÄ-Tests ist, dann ist die korrekte Bewertung 2 Punkte. Antworten, die den gleichen Inhalt haben, aber in anderen Worten wiedergegeben werden, bekommen auch 2 Punkte. Beispielsweise die Antwort: „Zwei Wochen" beim PRÄ-Test und „wir waren 14 Tage weg" beim POST-Test. Hier wird klar, dass der Patient die gleiche Information aus dem Gedächtnis abrufen kann, sich aber anders ausdrückt.

Die meisten Antworten dieser Kategorie bestehen nur aus einer Angabe und werden dadurch kaum die Teilleistung 1 Punkt erreichen. Die drei Fragen, die nur eine einfache Zahl abfragen sind: Alter des

Angehörigen, Anzahl der Urlaubstage auf der letzten Reise und Stockwerk des Arztbesuchs. Sollte die Antwort des POST- Tests nur eine Einheit entfernt von der Antwort des PRÄ-Tests sein, bekommt der Patient 1 Punkt. Zum Beispiel war die Antwort des PRÄ-Tests „14 Tage“ und beim POST-Test „15 Tage“.

Für die zwei Fragen, die eine Telefonnummer abfragen, gilt, dass der Patient 1 Punkt bekommt, auch wenn eine Zahl falsch war oder er die Postleihzahl vergessen hat.

Die Antwort der Frage nach dem Geburtstag des/der Angehörigen kann um einen Tag daneben liegen und bekommt trotzdem noch 1 Punkt. Die Antworten auf die beiden Fragen, die nach Monat und Jahr eines Events fragen können, sich um einen Monat unterscheiden und trotzdem 1 Punkt bekommen. Wenn ein Patient beispielsweise beim PRÄ-Test angegeben hat, er hätte seinen letzten Job im März 1966 verlassen und im POST-Test von April spricht, gibt das noch immer 1 Punkt.

Antworten beim POST-Test die einen wichtigen Aspekt wie den Tag des Monats des Geburtstags vergessen, oder die Telefonnummer mit mehreren Fehlern wiedergeben, bekommen 0 Punkte und werden als Gedächtnisverlust gewertet.

POST-Test Bewertung: Adressen

Die drei Fragen nach Adressen setzen sich aus mehreren Bestandteilen zusammen. Die Antwort beim POST-Test muss alle Bestandteile des PRÄ-Tests beinhalten und diese müssen auch inhaltlich identisch sein, um 2 Punkte zu erreichen. Wenn ein Bestandteil fehlt oder anders wiedergegeben wird, sollte eine Teilpunktezahl in Betracht gezogen werden. Jedes beim POST-Test neu genannte Bestandteil sollte in der Bewertung ignoriert werden.

Wenn ein Bestandteil fehlt oder falsch ist, wird eine Teilpunktezahl von 1 vergeben. Wenn mehr als ein Bestandteil fehlt und/ oder falsch ist, werden dafür 0 Punkte vergeben.

POST-Test Bewertung: Aufzählungen

Die sechs Aufzählungsfragen haben potentiell mehrere Antwortbestandteile. Eine Antwort des POST-Tests die die gleichen Elemente, wie beim PRÄ-Test aufzählt, bekommt 2 Punkte.

Wenn die Antwort beim PRÄ-Test keine Aufzählung war („Ich bin alleine verreist“) und die Antwort beim POST-Test wieder genau das gleiche besagt, werden auch hier 2 Punkte vergeben.

Wenn ein Bestandteil fehlt oder inkorrekt wiedergegeben wird, sollte man einen Teilpunkt in Erwägung ziehen. Alle beim POST-Test neu hinzugekommenen Details sollten in der Punktebewertung ignoriert werden.

Wenn ein Bestandteil beim POST-Test, verglichen zum PRÄ-Test, fehlt, wird dafür 1 Punkt vergeben. Auch wenn ein Bestandteil nicht richtig ist, wird 1 Punkt gegeben. Eine Aufzählung beim POST-Test bei der mehr als ein Bestandteil des PRÄ-Tests fehlt, wird mit 0 Punkten bewertet. In anderen Worten: wenn sich die Antwort des POST-Test signifikant von der des PRÄ-Tests unterscheidet, wird die Antwort mit 0 Punkten bewertet. Beispielsweise wurde beim PRÄ-Test von drei Reisebegleitern gesprochen und beim POST-Test behauptet man sei alleine verreist.

POST-Test Bewertung: Beschreibungen

Die sechs Beschreibungsfragen beinhalten potentiell mehrere Bestandteile und werden ähnlich wie die Adressen-/ Auflistungsfragen bewertet. Eine Antwort beim POST-Test, die eine ähnliche Beschreibung, zu der im PRÄ-Test abgibt, wird mit 2 Punkten bewertet. Wenn die Antwort des PRÄ-Tests beispielsweise war: „Wir sind nach Florida gereist, um der Hochzeit meiner Nichte beizuwohnen und um Urlaub zu machen“, wird dies als zwei Bestandteile gewertet (Hochzeit und Urlaub). Diese beiden Bestandteile sollten im POST-Test genannt werden um 2 Punkte zu erreichen. Jeder neue Bestandteil im POST-Test wird ignoriert.

Eine Antwort beim POST-Test mit mehreren Bestandteilen, jedoch einem fehlenden Bestandteil, verglichen mit dem PRÄ-Test, wird mit 1 Punkt bewertet. Um das Beispiel von oben weiter zu verwenden zählt eine Antwort wie: „Wir reisten nach Florida, um an der Hochzeit meiner Nichte teilzunehmen“, als 1 Punkt, da hier der Aspekt „Urlaub machen“ fehlt.

Eine Antwort beim POST-Test die keine vergleichbaren Details zum PRÄ-Test liefert gibt 0 Punkte.

ZUSAMMENFASSUNG DER POST-TEST BEWERTUNG

Nach der Auswertung des Interviews, sollte in jeder Spalte die nummerische Wertung von 0, 1, 2 angegeben worden sein. Diese Punkte werden nun zusammengerechnet und am Ende des Abschnitts eingetragen. Die Gesamtpunktzahl der sechs Teile werden dann am Ende des Fragebogens in die Tabelle eingetragen. Nun werden die sechs Teilbereiche miteinander addiert und anschließend wird diese Summe, durch die Summe aller Punkte des PRÄ-Tests, geteilt und mit 100 multipliziert.

Das Resultat ist der „Amnesie-Score“ und dieser zeigt ein Verhältnis des Erinnerungsvermögens VOR der Behandlung und NACH der Behandlung an.

„Amnesie - Score“:

$$(\Sigma_{\text{POST-Test}} / \Sigma_{\text{PRÄ-Test}}) \times 100$$

ANHANG 1

NUMMER DER FRAGE	KATEGORIE DER FRAGE	INHALT DER FRAGE
**Teil 1 Familienmitglied**		
**Frage 1**	Datum/ Zahl	Geburtstag (Monat/ Tag)
**Frage 2**	Datum/ Zahl	Alter
**Frage 3**	Adresse	Anschrift
**Frage 4**	Datum/ Zahl	Telefonnummer
**Frage 5**	Aufzählung	Mitbewohner
**Teil 2 Reise**		
**Frage1**	Datum/ Zahl	Reisetage
**Frage 2**	Name/ Ort	Name der Unterkunft
**Frage 3**	Aufzählung	Mitreisende
**Frage 4**	Beschreibung	Grund der Reise
**Frage 5**	Beschreibung	Schönes an der Reise
**Teil 3 Silvester**		
**Frage 1**	Aufzählung	Freunde
**Frage 2**	Name/ Ort	Ort des Abendessens
**Frage 3**	Name/ Ort	Ort des Abends
**Frage 4**	Beschreibung	Aktivität
**Frage 5**	Beschreibung	Aktivität um Mitternacht
**Teil 4 Geburtstag**		
**Frage 1**	Aufzählung	Freunde
**Frage 2**	Name/ Ort	Ort der Feier
**Frage 3**	Beschreibung	Aktivität
**Frage 4**	Aufzählung	Personen die beschenkten
**Frage 5**	Aufzählung	Erhaltene Geschenke
**Teil 5 Arbeit**		
**Frage 1**	Name/ Ort	Berufsbezeichnung
**Frage 2**	Name/ Ort	Name des Vorgesetzten
**Frage 3**	Adresse	Adresse des Arbeitsplatzes
**Frage 4**	Datum/ Zahl	Telefonnummer
**Frage 5**	Datum/ Zahl	Arbeitsbeginn (Monat/ Jahr)
**Teil 6 organische Beschwerden**		
**Frage 1**	Name/ Ort	Name des Arztes
**Frage 2**	Adresse	Adresse des Arztes
**Frage 3**	Datum/ Zahl	Stockwerk der Praxis
**Frage 4**	Beschreibung	Therapieplan
**Frage 5**	Datum/ Zahl	Symptombeginn (Monat/ Jahr)
